# Reconfigurable ADAMS Modeling of Localized Bearing Defects: Validation and the Role of Modeling Choices in Dynamic Response

**DOI:** 10.3390/s26206370

**Published:** 2026-10-09

**Authors:** Ori Schwarcz, Tal Bublil, Jacob Bortman, Shahar Shoham

**Affiliations:** Prognostic Health Monitoring Laboratory, Department of Mechanical Engineering, Ben-Gurion University of the Negev, P.O. Box 653, Beer-Sheva 8410501, Israel

**Keywords:** rolling-element bearings, localized bearing defects, multibody dynamics, MSC ADAMS, contact mechanics

## Abstract

Localized defects in rolling-element bearings produce vibration responses governed by contact events, load transmission, defect geometry, and system dynamics. To examine these mechanisms in a controlled and reconfigurable setting, this paper presents an MSC ADAMS framework for localized bearing faults, demonstrated on an SKF 6208 deep groove ball bearing with outer-ring spalls. The framework is evaluated against experimental test-rig measurements and a legacy 3D MATLAB bearing model, with healthy bearing contact forces checked against analytical load-distribution theory. The ADAMS model reproduces the spectral structure (including BPFO harmonics and selected modulation features), defect-interaction timing, and trends across defect sizes, though not absolute vibration amplitudes, while providing access to internal contact forces during defect interaction. This combination is used to examine two modeling issues that affect the mechanical interpretation of simulated bearing fault response. First, the outer-ring support definition controls the effective load path: adding support stiffness can transfer part of the applied load directly to the ground, reducing the load carried through rolling contacts. Second, the defect exit response depends on local contact formulation and geometric discretization. The legacy MATLAB model, based on a single-point Hertzian contact formulation, produces a sharper exit rebound, whereas the geometry-based ADAMS contact produces a more gradual return to the load zone. The study therefore contributes not only a validated simulation framework, but also mechanistic insight into how support and contact modeling choices shape the vibration and contact force responses used in bearing fault diagnostics, prognostics, and physical interpretation.

## 1. Introduction

Rolling-element bearings are fundamental components in rotating machinery, where cyclic loading, friction, lubrication conditions, and surface degradation can lead to wear and rolling contact fatigue [1,2]. Statistical bearing-life models provide useful population-level reliability estimates, but they cannot predict the exact onset of failure in an individual bearing [3]. This limitation motivates vibration-based condition monitoring. However, the measured diagnostic response depends not only on the presence of a defect but also on the system’s configuration, defect geometry, contact conditions, load distribution, wear evolution, and system dynamics. Dynamic modeling is therefore needed to reproduce diagnostic vibration features and to interpret the physical wear and contact mechanisms underlying these features [4,5].

Bearing defect modeling has developed from compact analytical descriptions toward increasingly detailed dynamic formulations. Review work has organized this literature into several major families, including periodic impulse train models, quasi-periodic impulse train models, nonlinear multibody dynamic models, and finite element models for localized and extended defects [6]. Early analytical models represented localized defects through periodic impulse or pulse-based excitation and related the resulting response to the bearing kinematics, load distribution, and spectral features [7,8]. Subsequent analytical formulations introduced finite-width defect pulses and examined the effects of radial and axial loading [9]. Later nonlinear time-domain analytical and discrete lumped-parameter models introduced richer descriptions of contact and defect interaction. Across these studies, the modeled mechanisms include Hertzian contact, internal clearance, time-varying stiffness, cage slip, extended spalls, and load redistribution during defect interaction [10,11,12,13,14]. In parallel, commercial multibody environments and custom numerical formulations were used to represent bearings as system components. Examples include six-degree-of-freedom bearing formulations, solid-to-solid contact models, detailed cage dynamics, angular domain descriptions, flexible rotor bearing models, and models for different bearing architectures and fault conditions [15,16,17,18,19,20,21,22,23].

Although these developments provide a strong modeling foundation, several issues remain important when simulated bearing fault response is interpreted physically rather than used only as a signal source. Wear evolution can involve interacting mechanisms that reshape contact conditions and surface topography over time [4], while localized defect shape and contact profile can influence the generated pulse waveform and contact deformation [24]. Rolling contact fatigue also involves local, multiaxial, and non-proportional stress histories that are difficult to reduce to a single simplified description [25]. Within dynamic bearing models, predicted responses can depend on clearance, coupled rotor-bearing fault interactions, and assumptions about rolling-element inertia, slippage, cage-related interactions, and lubrication. These issues are addressed in studies that either explicitly model selected effects or examine the consequences of simplifying them [26,27,28]. At the defect scale, simplified regular defect geometries may change the predicted force and vibration response relative to irregular or more realistic defect shapes [29]. Local edge characteristics, together with the distinction between entry and exit events, can also influence the resulting force wave and vibration characteristics [30]. These observations indicate that simulated bearing fault responses should be interpreted with attention to the modeling choices and simplifications involved. Recent work further shows that bearing fault simulation is advancing along several complementary directions. At the local defect scale, Zhang et al. [31] showed that defect edge geometry and irregular raceway morphology can influence rolling-element impact behavior and high-frequency vibration response. At the system scale, Shafiee et al. [32] integrated ADAMS dynamic bearing models with finite-element rotor and housing representations, emphasizing the role of rotor flexibility, support deformation, clearance, and bearing–housing interaction in bearing dynamic response. ADAMS-based studies have also been used directly for bearing fault signal simulation, from simplified inner and outer race crack models [33] to experimentally compared inner race fault simulations evaluated through statistical indicators [34], and to a simplified three-dimensional MSC Adams ball-bearing model used to generate synthetic vibration signals for inner and outer race defects [35]. Accordingly, the objective of this paper is to establish the validity and contribution of a reconfigurable MSC ADAMS framework for localized bearing-defect simulations. The contribution of the paper is threefold. First, the framework is demonstrated for an SKF 6208 deep-groove ball bearing with outer-ring spalls and is validated against experimental test-rig measurements, the laboratory MATLAB bearing model, and an analytical healthy-bearing contact-force reference. Second, the validation is used to show that the outer-ring support definition can change the effective load path through the bearing: when support stiffness is added, part of the applied load is transferred directly to the ground rather than through the rolling contacts, making the required support calibration dependent on defect size. Third, the model is used to analyze defect entry, defect-floor impact, and defect exit through internal contact-force outputs, showing that the physical interpretation of these events depends on the contact formulation and geometric discretization used in the simulation. The paper first describes the ADAMS bearing model, the simulation procedure, and the validation references. It then examines the support and contact-modeling choices before presenting vibration and contact-force comparisons in the time, order, cycle, and contact-force domains, with additional validation and sensitivity results provided in the appendices. Finally, the discussion defines the conditions under which the framework can be used as a mechanically interpretable basis for future diagnostic, prognostic, and mechanistic bearing-fault studies.

## 2. Model and Validation Setup

This section presents the ADAMS modeling framework, the main assumptions and constraints, the simulation procedure, and the validation references used in this study. MSC ADAMS is used as a multibody simulation environment for representing the motion and contact interactions of the bearing components. The simulated system is an SKF 6208 deep-groove ball bearing with an outer-ring defect. Although this paper focuses on that configuration, the modeling framework is designed to be reconfigurable for other bearing geometries and defect types, although those configurations are not validated in the present study. A research paper using this model for different fault conditions has already been published [36], demonstrating the model’s reconfigurability and showing a correlation between the internal dynamics of the bearing and the proposed CIs for an angular contact bearing with a ball defect operating under combined radial and axial load.

Accurate representation of rolling-element interaction with a local defect requires high temporal resolution and can be computationally demanding. To manage this computational requirement, the simulations were carried out using a workflow controlled with MATLAB R2024a, in which MATLAB managed simulation execution, data handling, memory release between simulation stages, and parallel runs. Meanwhile, ADAMS was used for the multibody model and dynamic calculations. The simulations presented in this paper used a time step of 5 × 10^−8^ s, which was originally selected to match the resolution requirement of the legacy MATLAB model. Throughout the paper, the proposed model is referred to as the “ADAMS model”, while the earlier in-house benchmark model is referred to as the “MATLAB model”. The main text focuses on the comparisons and modeling analyses required to support the central claims of the paper. Meanwhile, the appendices provide complementary validation and sensitivity checks, including healthy-bearing contact-force benchmarking, cycle-domain defect-interaction analysis, sensitivity to load and defect-position configuration, and time-step sensitivity.

### 2.1. Model Setup

All bearing components were modeled as rigid bodies: the outer and inner rings, balls, cage, and shaft. The balls and cage were left unconstrained in all six degrees of freedom, so their motion was governed by contact interactions, gravity, and the prescribed system constraints. The clearance between the balls and the cage holes is set to 3% of the ball radius. These modeling choices were made to allow the dynamics to be dictated primarily by the geometry of the defect, rather than by prescribed rolling-element motion, constant angular spacing between balls, or predefined defect-displacement functions. Contacts are only defined between the balls and other bodies to avoid possible contact between the rings themselves or between the cage and either ring. Normal contact uses the ADAMS Impact formulation with stiffness values derived from Hertzian contact theory, and tangential contact uses the ADAMS Coulomb formulation (parameters listed in Table 1). The contact stiffness values were calculated from the geometry and material properties of the contacting bodies using the Hertzian contact formulation. The remaining IMPACT and Coulomb parameters were retained as fixed model-level contact settings throughout the simulations rather than calibrated to the experimental vibration response. The force exponent of 2.2 and the 0.01-mm penetration depth are consistent with standard ADAMS IMPACT settings, while the damping and friction parameters were kept fixed after preliminary sensitivity testing to provide stable and consistent contact behavior across all simulated cases. Friction is applied at the ball–raceway contacts only and was not applied to the cage in order to allow it to serve mainly as a spacing element and reduce unnecessary contact effects.

**Figure 1:**
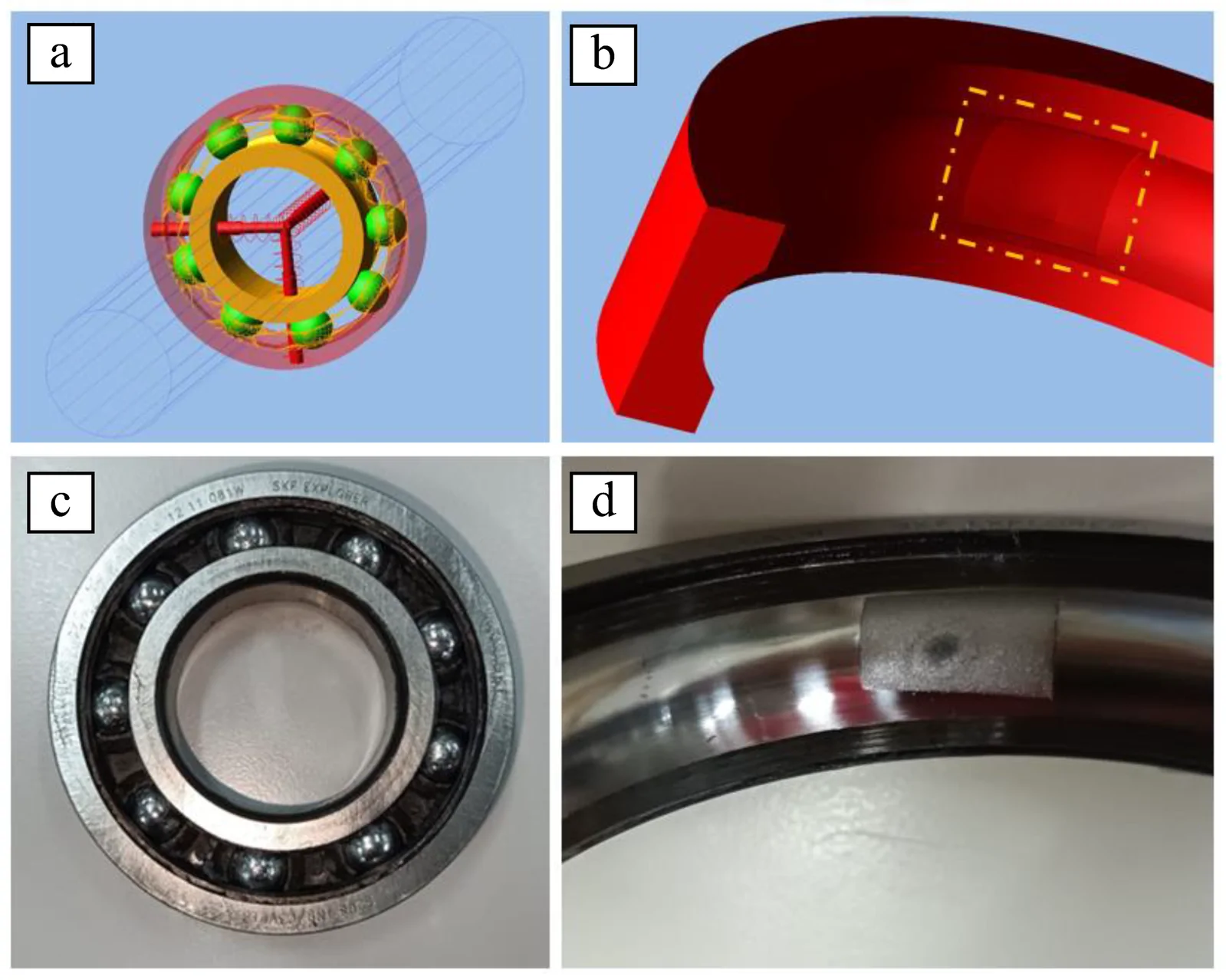
(**a**) ADAMS model of the bearing assembly. To expose the internal geometry, the outer ring is rendered transparent red, the cage as a gold wireframe, and the shaft as a blue wireframe. (**b**) Close-up of the outer ring only, showing an 11-mm spall defect. The defect location is highlighted by a yellow dashed rectangle. (**c**) Photograph of an SKF 6208 bearing used in the experiments. (**d**) Close-up of the same bearing’s outer ring, showing a 12-mm artificial spall defect.

As a compromise between geometric accuracy and computation time, curved bodies are represented as polygons built with 20,000 faces. This discretization can introduce numerical noise into the simulated signals but substantially reduces runtime compared to the Parasolid option available in ADAMS, which preserves curved features. Figure 1 presents the ADAMS model in the top row, along with pictures of a defective bearing used in the experiment: (a) shows the full model assembly where the shaft and the cage were drawn in wireframe to allow viewing the inner part more clearly, (b) shows a close-up view of the defect whose location is marked within a yellow dashed frame, while (c) and (d) show a tested bearing and its defective region, respectively.

The spall defect was constructed by intersecting a torus with a box and then subtracting the resulting volume from the ring surface. This method ensures a defect with constant depth governed by the torus minor radius, which was set to be 0.1 mm larger than the groove radius. The torus major and minor radii were set to 29.635 mm and 6.519 mm, establishing an outer raceway contact radius of *r* = 36.154 mm. The box defines the defect length and width, so the specified defect length in the ADAMS geometry corresponds to a chord length. In the MATLAB model, the same nominal defect-length input corresponds to the arc length along the outer raceway. Thus, the same nominal values define slightly different physical lengths in the two models. For the outer-raceway radius of 36.154 mm used here, arc lengths of 5 mm, 11 mm, and 17 mm correspond to chord lengths of 4.996 mm, 10.958 mm, and 16.844 mm, respectively. Compared with the corresponding ADAMS chord inputs of 5 mm, 11 mm, and 17 mm, the resulting geometric discrepancies are approximately 0.08%, 0.39%, and 0.92%, respectively, and therefore remain below 1% over the examined range. The box defines the defect length and width, which means those sizes are measured for ADAMS simulations as chord length, unlike the arc-length measurements used in the experimental and MATLAB model baseline results. This mismatch creates an error for large defect lengths; however, in this study, the resulting discrepancy is negligible, with the maximum error being under 1%.

### 2.2. Model Assumptions and Constraints

This subsection describes the relations and constraints defined between the bodies modeled in the ADAMS bearing. A summary of all degrees of freedom assigned to the components is presented in Table 2. The inner ring was rigidly fixed to the shaft, with no relative motion permitted. The shaft was constrained to rotate only about its axis, with all translational and off-axis rotational degrees of freedom suppressed. Rotation was imposed through the ADAMS joint motion element, which prescribes angular velocity directly, and no torque was applied. For the outer ring, the boundary conditions were chosen to reproduce the intended loading while keeping the load path through the bearing itself.

**Figure 2:**
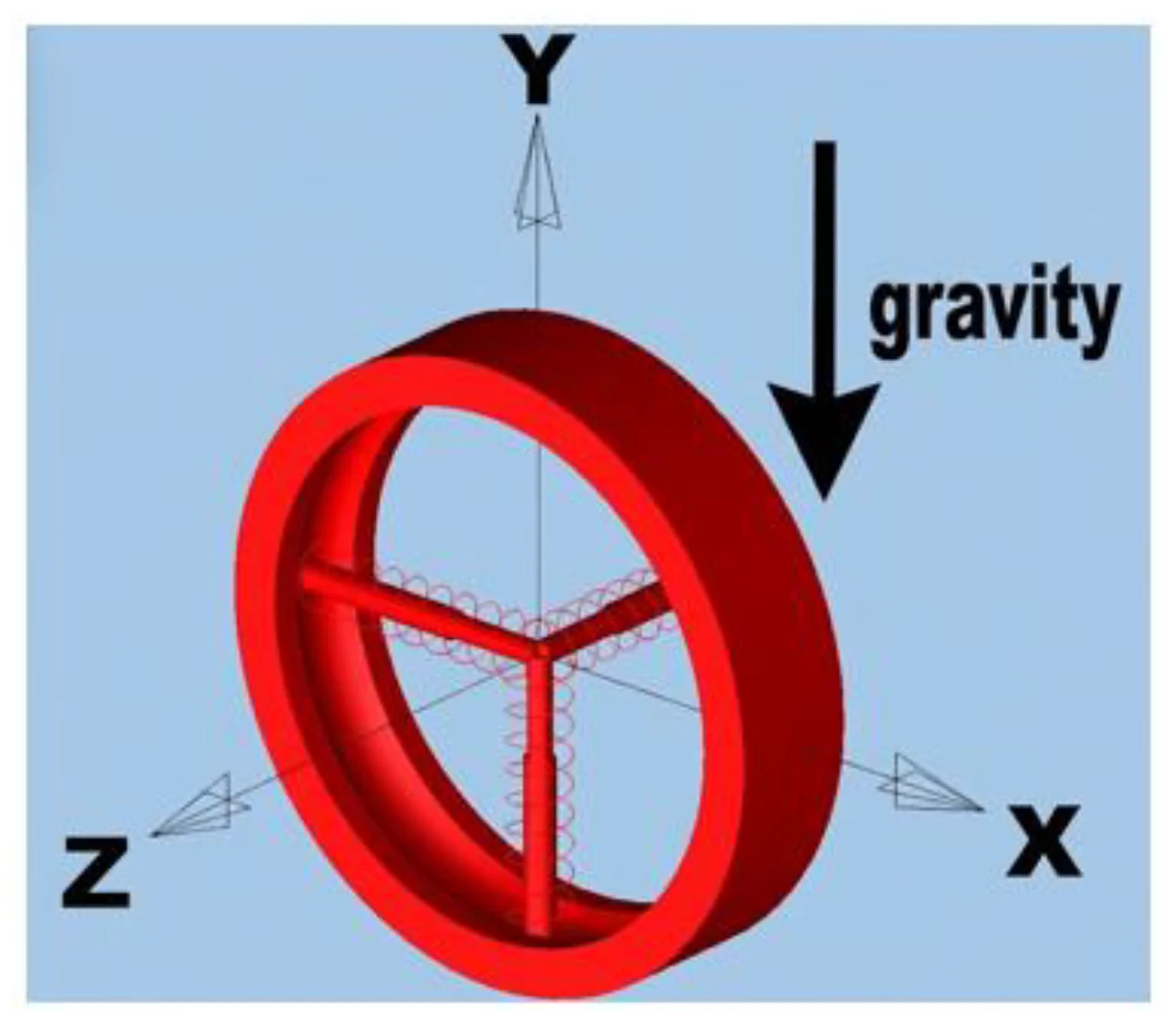
Damper configuration showing radial and axial attachment points relative to the defined coordinate system and gravity direction.

A purely radial external force was applied to the center of mass of the outer ring through an ADAMS force element. The reaction was applied to ground, which allowed the force direction to be defined directly as a 3D vector. For this study, the force was aligned with gravity, and the spall was placed at the top of the outer ring, that is, at the 12 o’clock position in the polar view. The outer ring was constrained against rotation about all three axes, while its translational motion was retained in the X, Y, and Z directions. These translational directions were connected to ground through three independent dampers aligned with the principal coordinate directions shown in Figure 2. Springs were excluded from the translational outer-ring support because their inclusion caused an unwanted force leakage from the outer ring to the ground without passing through the bearing itself.

This modeling approach and its justification are examined further in Section 3.1 through the support-stiffness sensitivity analysis, and the damping-value selection is discussed in the same section based on the healthy-bearing damping study.

Each damper was modeled as a translational element with separate attachment points on the ground and on the outer ring. The ground-side endpoint of all three dampers was placed at the geometric center of the bearing, coincident with the shaft axis and the inner-ring center. The ring-side endpoints of the radial dampers (X and Y) were placed on the outer surface of the outer ring, at mid-width. The axial damper (Z) was connected to a virtual point aligned with the ring center and treated as part of the rigid outer-ring body. This avoided unwanted rotational coupling while still constraining axial motion.

### 2.3. Simulation Procedure

The dynamic simulation was structured into three sequential stages: initiation, convergence, and steady-state. Operating conditions, including shaft speed and radial load, were active from the start of the simulation at *t* = 0. This division into stages was used only to separate the initial settling process from the stable steady-state data-acquisition regime. During the initiation stage, the components transitioned from their idealized geometric positions to their dynamic working positions. To simulate this adjustment efficiently, a variable time-step strategy was used. The sequence began with a time step of 10^−6^ s and was then incrementally reduced to the target high-resolution value of 5 × 10^−8^ s. This process spanned a total physical duration of approximately 0.53 s.

The convergence phase followed the initiation stage and was used to remove residual transient effects. During this phase, the time step was fixed at 5 × 10^−8^ s. To ensure a stable periodic regime, a conservative duration of 3 × 10^6^ time steps was used, corresponding to 0.15 s. The final stage was the steady-state run, which used the same high-resolution time step and had no fixed upper limit on the total number of time steps. Its duration was determined by user-defined conditions, typically the required number of shaft revolutions at the configured speed. Only this steady-state portion was retained for signal analysis and order-domain processing, ensuring that the analyzed results reflected stable dynamic behavior.

### 2.4. Reference Baselines

This section presents the reference baselines used in this study to validate the proposed ADAMS model. The two main reference baselines are the legacy MATLAB bearing model earlier developed in the laboratory and the experimental test rig. An analytical reference was also used to validate the contact-force distribution for a healthy bearing simulation. This validation step is presented in Appendix A, while the analytical formulation is based on [37] and presented in Appendix B. A complementary cycle-domain comparison of defect-interaction timing, including entry, floor-impact, and exit events for the selected defect cases, is provided in Appendix C.

#### 2.4.1. Legacy MATLAB Benchmark

In 2014, Kogan, Klein, and Bortman, along with collaborators from the Laboratory for Mechanical Health Monitoring in the Department of Mechanical Engineering at Ben-Gurion University of the Negev, developed a 3D dynamic model of Single and duplex bearing configurations, including angular-contact and deep-groove ball bearings, implemented in a MATLAB environment [38]. The model was limited to simulating localized spall defects on both the inner and outer rings and was validated with analytical calculations and experimental results. This model has served as the laboratory’s simulator benchmark for subsequent fault-diagnosis studies and as one of the main validation references in the present study.

#### 2.4.2. Experimental Reference System and Signal Processing

**Figure 3:**
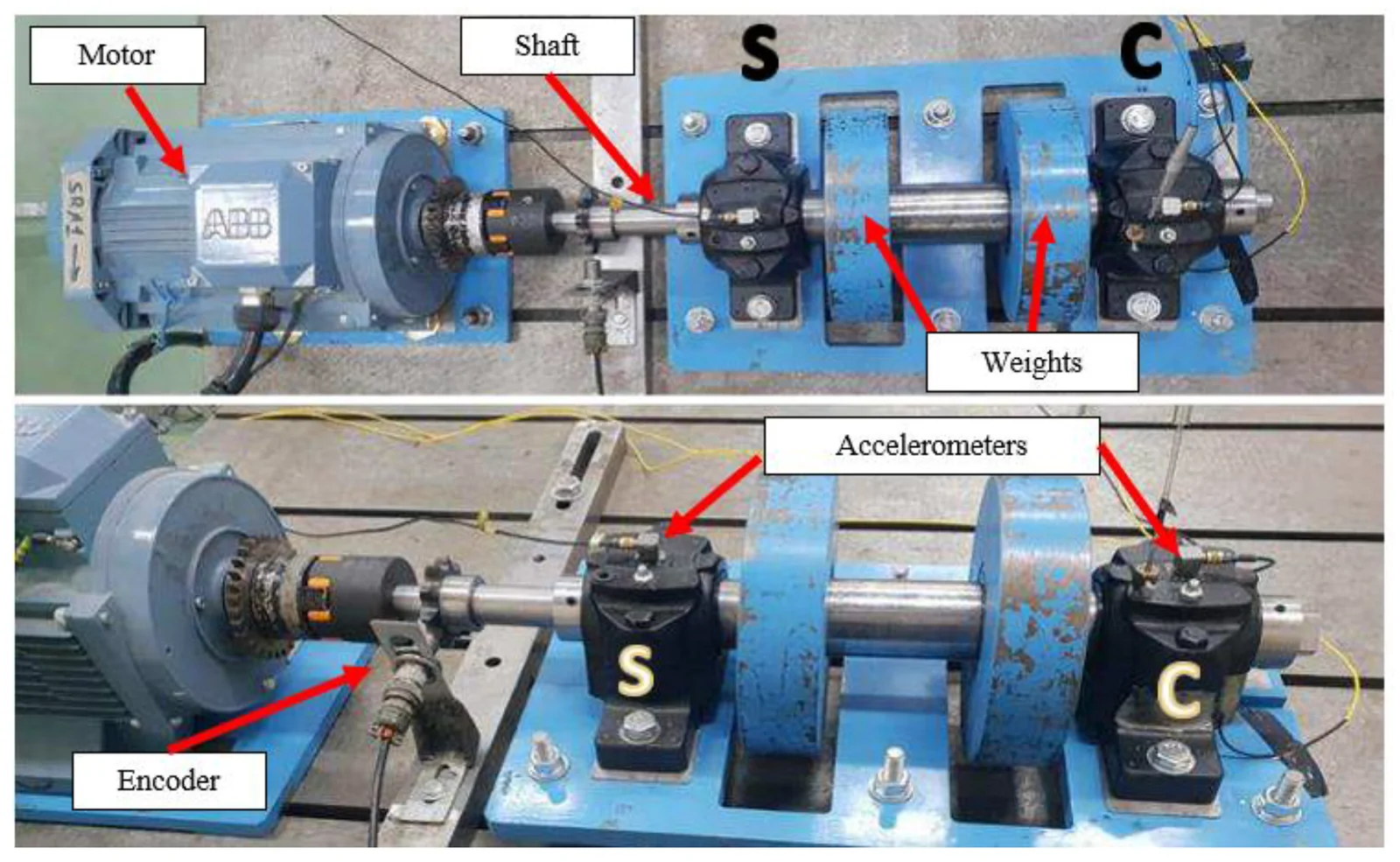
Experimental test rig used for bearing validation [39]. The checked bearing (C) contains an artificial outer-ring spall and is mounted alongside a support bearing (S) on a shared shaft. Radial load is applied using suspended weights aligned with gravity. A rotary encoder tracks shaft speed, and tri-axial accelerometers mounted on each bearing block capture directional vibrations.

The experimental reference system is shown in Figure 3, while a defective bearing was previously shown in Figure 1. The test rig consists of an electric motor, a flexible coupling, and a shared shaft supported by two identical bearings. The tested bearing, labeled C, is an SKF 6208 deep-groove ball bearing (SKF, Gothenburg, Sweden; supplied by EMCOL Ltd., Ashdod, Israel) with an artificial outer-ring defect produced by electrical discharge machining, while a second identical bearing, labeled S, serves as the support bearing. Seventeen defects were fabricated, with lengths from 1 mm to 17 mm in 1 mm increments, and the shaft was driven at a constant speed of 40 RPS (2400 RPM). A 200 N radial load was applied to the shaft through suspended weights. In the rig, the load passes through the shaft and inner rings, so the loaded zone is at the bottom of the checked bearing. The defect was therefore placed at the bottom of the outer ring to force rolling-element interaction inside the loaded zone.

The same bearing geometry, nominal defect-length inputs, shaft speed, and load magnitude were used in the ADAMS and MATLAB simulations. However, the ADAMS configuration placed the defect and loaded zone at the top of the bearing, whereas in the experimental and MATLAB references they were positioned at the bottom. The effects of this positioning are shown to be negligible in Appendix D for the case studies examined in this paper, where ball inertia effects are much greater than gravity effects. At lower rotation speeds, however, gravity may have a more meaningful effect. Tri-axial accelerometers were mounted on the housings of both bearings and placed directly above the bearings. The sensor axes were aligned with the primary physical directions of the setup: vertical (gravity/load), horizontal (radial), and axial (along the shaft). Shaft rotation was monitored by a magnetic rotary encoder mounted on the drive shaft, which provided angular position data for speed tracking and for resampling the vibration signals with respect to shaft position. To suppress variability caused by thermal transients, vibration data were recorded only after the rig reached a thermal steady state.

### 2.5. Validation Protocol

The validation strategy was designed to evaluate the ADAMS model from both observable and internal dynamic perspectives. The main text focuses on the comparisons most directly related to the main objectives of the paper: spectral agreement with experimental measurements, comparison with the legacy MATLAB model, and contact-force analysis during defect interaction. Additional validation and sensitivity checks are provided in the appendices, including healthy-bearing contact-force benchmarking, cycle-domain defect-interaction analysis, and sensitivity to load and defect-position configuration. This structure keeps the main text focused on the modeling choices and their physical implications, while retaining the supporting validation evidence needed to assess the model more broadly. A concise comparison of the experimental reference, legacy MATLAB benchmark, and ADAMS model is provided in Table 3.

Two defect cases, with spall lengths of 5 mm and 11 mm, are used for detailed analysis of contact-force behavior and defect-interaction stages. A wider spectral comparison is then provided across all 17 defect cases to evaluate whether the main BPFO-related spectral structure is preserved over the full defect-length range. Together, these comparisons are used to assess whether the ADAMS model reproduces the main observable characteristics of the defect-bearing response while also providing mechanically interpretable internal quantities.

The signal-processing procedure was intentionally kept minimal. The experimental signals were resampled using the shaft-speed measurement from the rotary encoder, transforming the data from the time domain to the cycle domain, where one cycle corresponds to one shaft rotation. The simulation signals did not require resampling because the prescribed shaft speed was constant. For spectral analysis, the cycle-domain signals were transformed to the order domain using FFT, so that periodic events are represented by their occurrence rate per shaft rotation rather than by frequency in time. The FFT length was selected using power-of-two sample blocks. The order-bin spacing was determined by the ratio between the number of samples per shaft revolution and the FFT length. For the simulation spectra, 40 RPS and a time step of 5 × 10^−8^ s correspond to 500,000 samples per revolution; using a 2^22^-sample FFT therefore gives an order-bin spacing of 500,000/2^22^ = 0.1192 orders. The experimental signals were first resampled with respect to shaft position, and the corresponding cycle-domain sampling density and FFT length yielded an order-bin spacing of 0.0078 orders. The characteristic bearing frequencies, expressed in orders, were calculated analytically as BPFO = 3.59, BPFI = 5.41, BSF = 2.37, and FTF = 0.40. In addition, signal alignment consisted only of horizontal shifting and was used only to improve visual comparability between sources and did not modify the underlying signal content.

## 3. Dynamic Modeling Considerations and Mechanistic Insights

Two modeling choices implemented in ADAMS were found to have a direct influence on the predicted system physics: the support architecture of the outer ring and the local contact formulation at the defect. This section first motivates the damping value selected for the outer-ring support, then examines why support stiffness was omitted, and finally analyzes the resulting contact forces during defect interaction.

### 3.1. Outer-Ring Support

**Figure 4:**
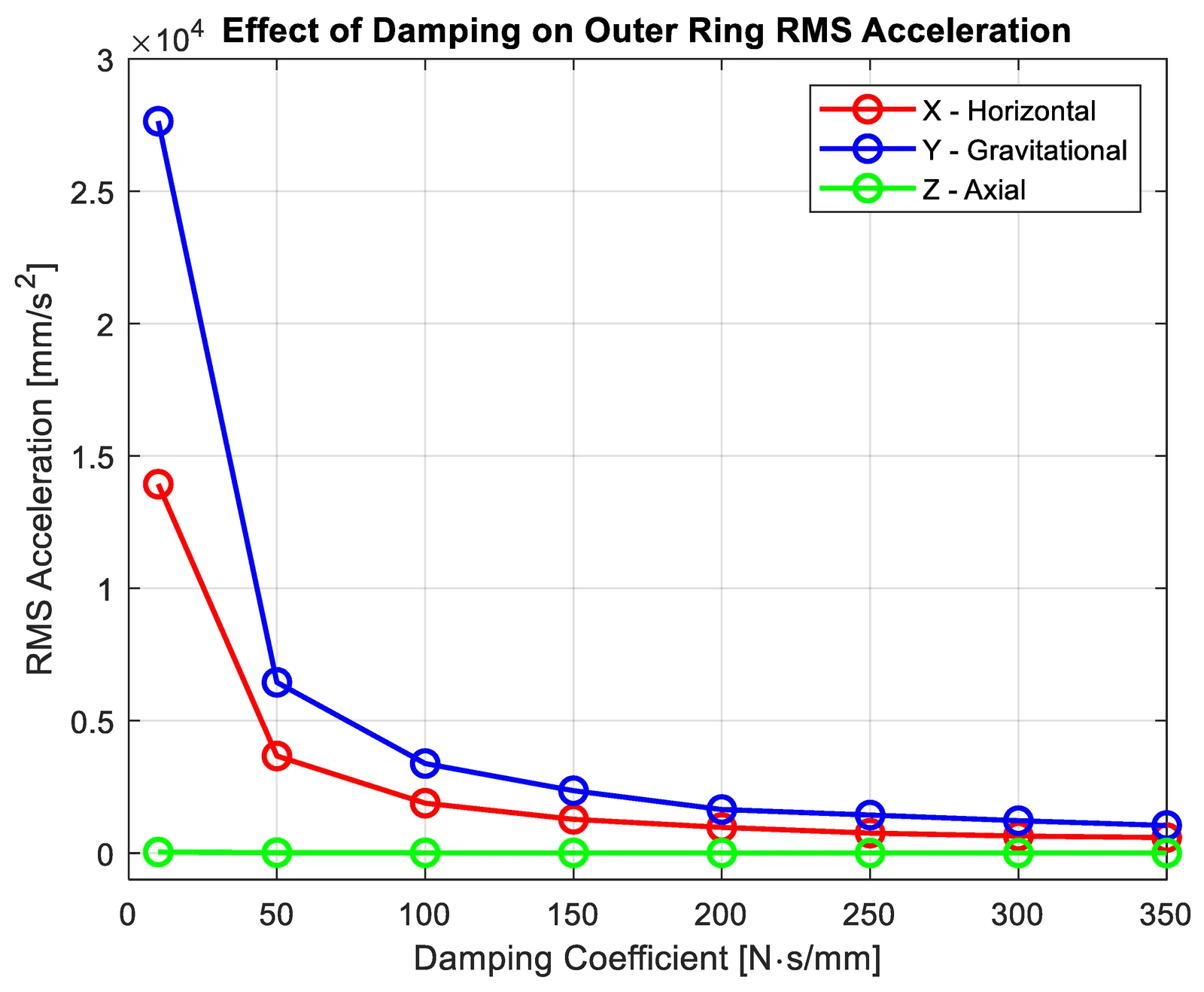
RMS acceleration of the outer ring in a healthy bearing under a 200 N load applied in the direction of gravity.

The outer-ring damping value was selected from healthy-bearing baseline simulations under a 200 N radial load applied in the vertical direction. Figure 4 shows the resulting RMS acceleration of the outer ring in the three principal directions. The horizontal and vertical (gravitational) responses decrease rapidly as damping increases up to 200 N·s/mm and then approach stable values. The axial response remains low over the full range. Based on this behavior, 200 N·s/mm was adopted as the standard damping value. This value is high enough to stabilize the main directional responses while still keeping the initiation stage reasonably short. The selected coefficient is therefore treated as a model-level support parameter chosen to obtain a stable outer-ring response with a practical initiation time, rather than as an estimate of the damping properties of the experimental support.

To justify the use of damping-only support for the outer ring, a sensitivity analysis was carried out on one healthy case and two defective cases, with 5-mm and 11-mm outer-race spalls. The damping coefficient was fixed at 200 N·s/mm, and the support stiffness was varied from 0 to 5000 N/mm.

**Figure 5:**
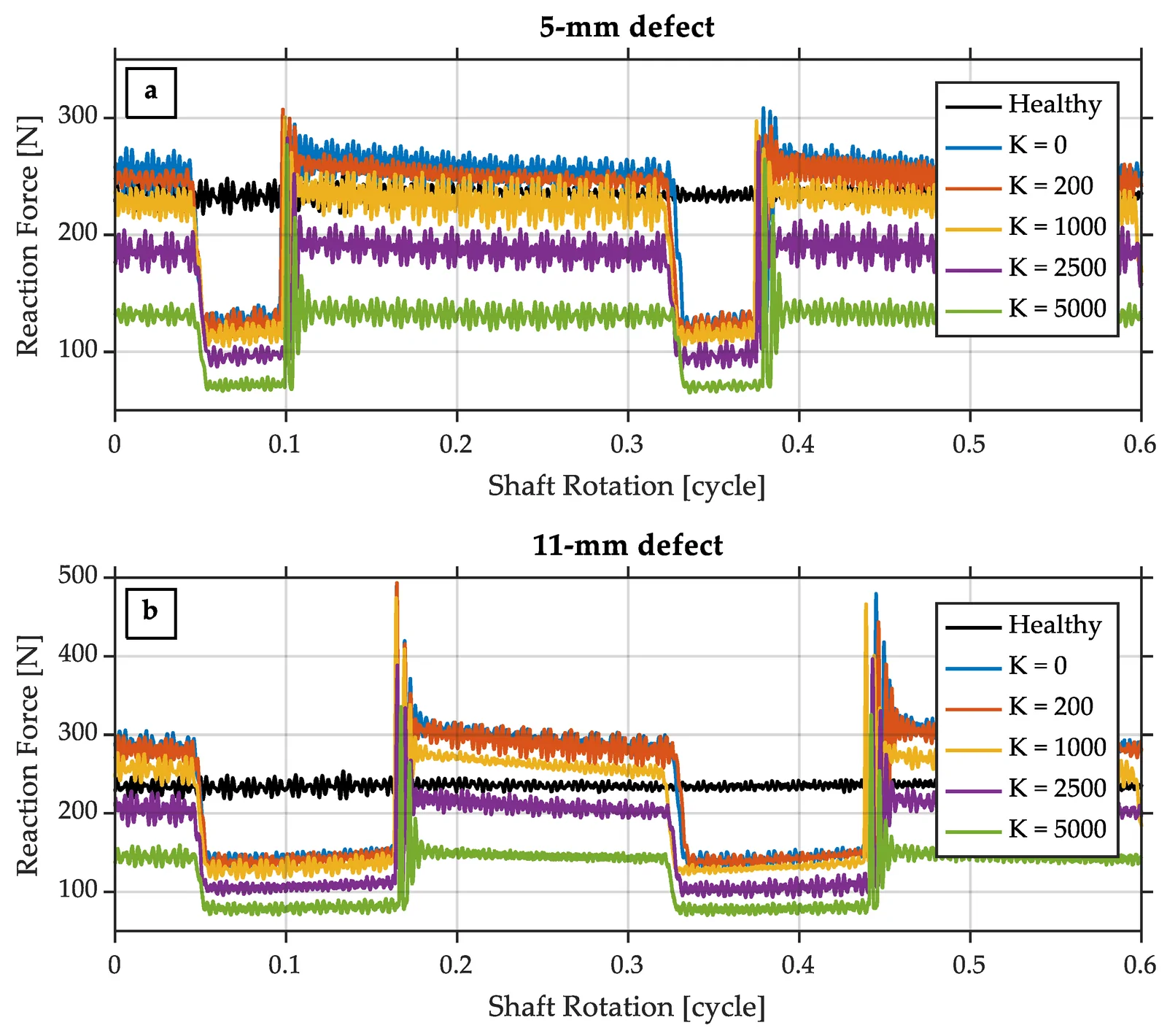
Reaction force at the shaft constraint vs. cycle for (**a**) 5-mm and (**b**) 11-mm defects. Each subplot compares the healthy baseline (black) against defective simulations with varying support stiffness.

Figure 5 shows the reaction force at the shaft constraint that connects it to the ground over a 0.6-cycle window. This force represents the net load transmitted through the bearing. In the healthy case, it stabilizes around 235 N, which matches the applied 200 N radial load plus the weight of the assembly, about 3.3 kg, acting in the same direction. In the defective cases, the force drops when a rolling element passes through the spall. This drop is physically consistent with the temporary loss of load support during defect interaction, and it agrees qualitatively with the load-cell behavior reported by Bublil et al. [40].

The main result of Figure 5 is not the force drop itself, but the downward shift in the full reaction-force signal as stiffness increases. As stiffness is added to the support, the reaction force measured through the bearing decreases. This indicates that an increasing part of the applied load is carried directly to the ground through the springs, bypassing the rolling contacts. The mean reaction force was calculated for each configuration to quantify this leakage effect. To isolate the steady-state behavior from transient entry and exit effects, the force was averaged over the specific reaction-force ranges defined in Table 4. For the defective cases, separate means were calculated for the “High Range”, where the rolling element is outside the spall, and the “Low Range”, where the rolling element is inside the spall.

**Figure 6:**
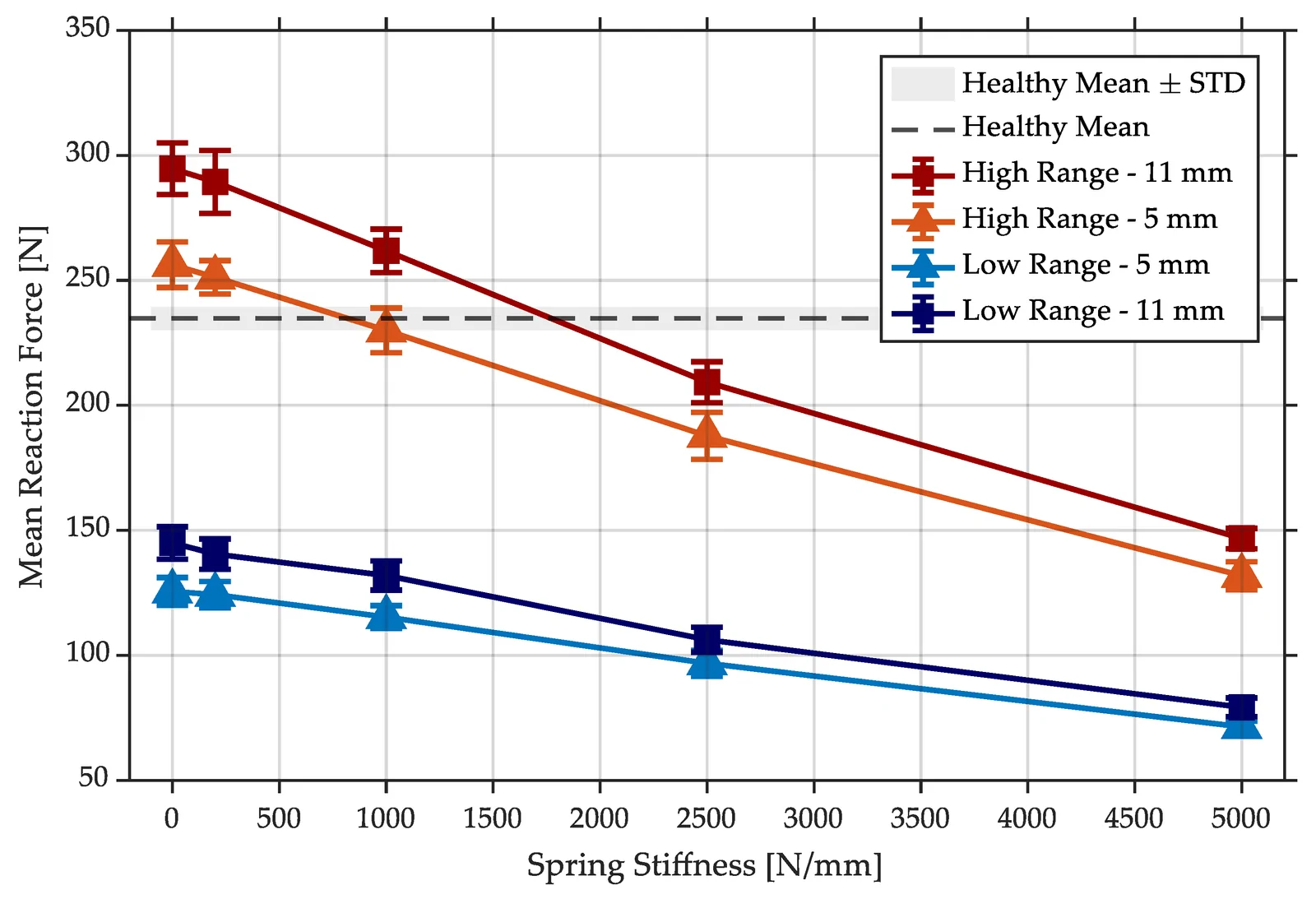
Mean reaction force as a function of support stiffness for the healthy case and for the 5-mm and 11-mm defect cases.

Figure 6 shows the leakage effect in aggregated form by plotting the mean reaction forces as a function of support stiffness. The results show an approximately linear decrease in mean force as support stiffness increases. This provides further evidence of load leakage: as stiffness rises, an increasing part of the external load no longer passes through the rolling contacts, so the bearing operates under a lower effective load than intended.

In principle, this effect could be compensated by tuning the stiffness value so that the post-transient out-of-spall reaction force in the defective case matches the healthy-bearing baseline force. However, as shown in Figure 6, the required stiffness also depends on defect size. For the 11 mm defect, the intersection with the healthy baseline occurs near 1800 N/mm, while for the 5 mm defect, it occurs near 1000 N/mm. A spring-damper support would therefore require case-specific retuning whenever the defect geometry changes. Such compensatory tuning would defeat the purpose of maintaining a uniform simulation architecture, because it would make it difficult to decouple the influence of defect geometry from the effects of support calibration.

### 3.2. Contact Formulation and Defect-Exit Physics

**Figure 7:**
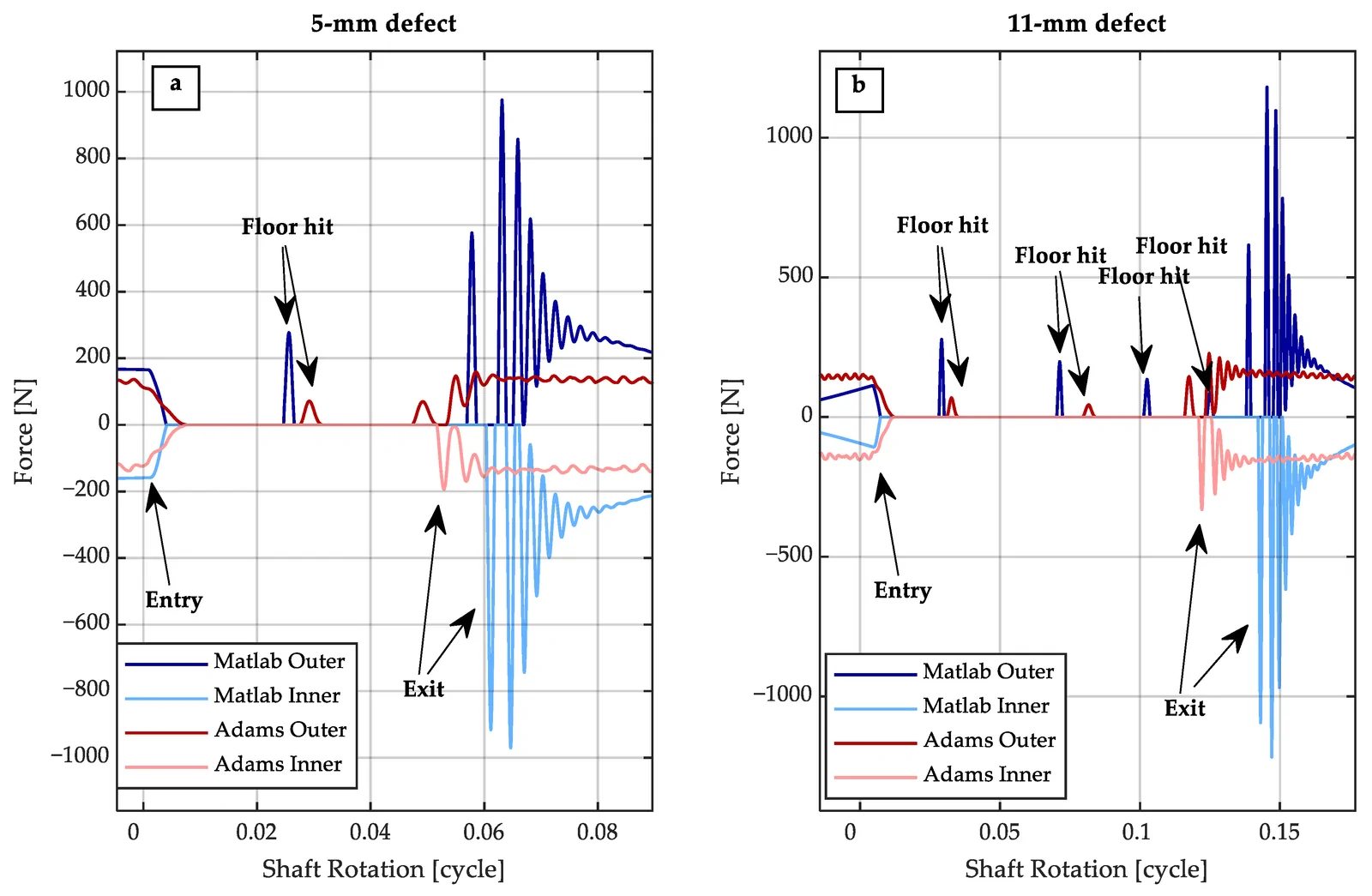
Contact force response during ball-to-defect interaction for (**a**) 5-mm and (**b**) 11-mm spall lengths.

Contact forces are central to the vibration behavior, fatigue life, and spall propagation of rolling-element bearings. In the present study, these forces provide internal dynamic information that is not available directly from the experimental acceleration measurements. Figure 7 presents the contact force over the inner and outer rings during defect interaction in both models. Subplot (a) corresponds to the 5-mm defect and subplot (b) to the 11-mm defect.

For both defect lengths, the ADAMS results exhibit higher pre-interaction fluctuations than the MATLAB results, while the MATLAB contact-force signals are smoother. During defect-floor contact, MATLAB shows higher peak forces and more repeated impacts. Despite these differences, the contact-force responses in both models follow the same general interaction sequence as the rolling element passes through the spall:

- Defect entry: Both models show a sharp drop in contact force, consistent with the ball entering an unsupported region.
- Mid-defect motion: Depending on the spall geometry, rotation speed, and load, the ball may impact the defect floor one or more times. ADAMS captures these impacts as distinct force peaks.
- Defect exit: In the MATLAB model, a sharp rebound impact is followed by high-magnitude oscillatory peaks that are attributed to repeated ball–ring impacts. In ADAMS, the exit behavior is smoother. Small oscillations may appear, but they quickly converge toward the steady-state load, suggesting a more gradual reintegration into the load zone.

A quantitative comparison of the contact-force response at key phases of the defect interaction is presented in Table 5. These include the contact-force level just prior to entering the defect, the force magnitude during floor impact, and the maximum contact force at the exit. The values are extracted from the single defect interaction shown in Figure 7. Pre-interaction and floor-impact values refer to the ball-to-outer-ring contact, whereas the exit maximum is the largest force magnitude observed across the simultaneously plotted inner- and outer-ring contact responses.

During development of the ADAMS model, several possibilities were examined to explain the discrepancy in the exit stage. These included restoring the outer-ring support stiffness, tuning contact-force parameters, and introducing a spring-damper coupling between the inner ring and the shaft. However, none of these adjustments reproduced the MATLAB interaction profile without distorting the overall bearing dynamics. The cage architecture was also investigated as a possible contributor to the difference. Because the MATLAB model enforces a constant angular spacing between rolling elements, the free-body dynamics and clearance of the ADAMS cage were examined. The hypothesis was that the absence of a rigid angular constraint in ADAMS might allow the ball to interact more softly with the defect exit, remaining in position until the cage clearance closed and pushed it out. However, internal dynamic inspection showed that this clearance gap closes before the ball reaches the exit, so cage clearance does not explain the lower ADAMS exit forces.

The main difference is therefore consistent with differences in the contact formulations. In the MATLAB simulation, contact is modeled using a single-point Hertzian formulation applied uniformly along the raceway. This formulation assumes a nominal contact stiffness based on smooth raceway curvature, without resolving the local geometry at the defect edge. Such an approach is suitable for smooth contact regions, but it becomes less appropriate near sharp geometric discontinuities, where the assumptions of continuous curvature and purely elastic Hertzian contact are no longer satisfied. As the effective local radius decreases, the resulting contact stiffness can become highly sensitive to the assumed local curvature if the same nominal Hertzian formulation is applied at the edge. This may contribute to the sharp rebound force generated by the MATLAB model at the defect exit.

In contrast, ADAMS uses a multipoint-contact formulation that calculates geometric overlap between faceted surfaces at each time step. Only regions undergoing actual contact contribute to the normal force, so the response depends on the local geometry involved in the contact event. The resulting ADAMS exit response is therefore less dominated by a single sharp rebound event and more strongly governed by the resolved geometry of the transition back into the load zone.

The higher baseline fluctuations observed in the ADAMS contact forces prior to defect interaction are consistent with this discretized geometry. Modeling the ball surface as a polygon mesh with 20,000 faces introduces micro-edges that increase sensitivity to local contact variations. A dedicated faceting-sensitivity analysis is provided in Appendix E, including the effects of surface resolution on the principal defect-interaction forces and on the characteristic defect-exit response. Taken together, these findings indicate that the multipoint ADAMS formulation provides a more geometry-dependent representation of the defect-exit transition. They also show that contact-force outputs cannot be interpreted independently of the contact formulation that generated them. Because defect-scale contact forces were not measured experimentally, this comparison does not establish that either contact formulation is more physically accurate.

## 4. Spectral Validation

**Figure 8:**
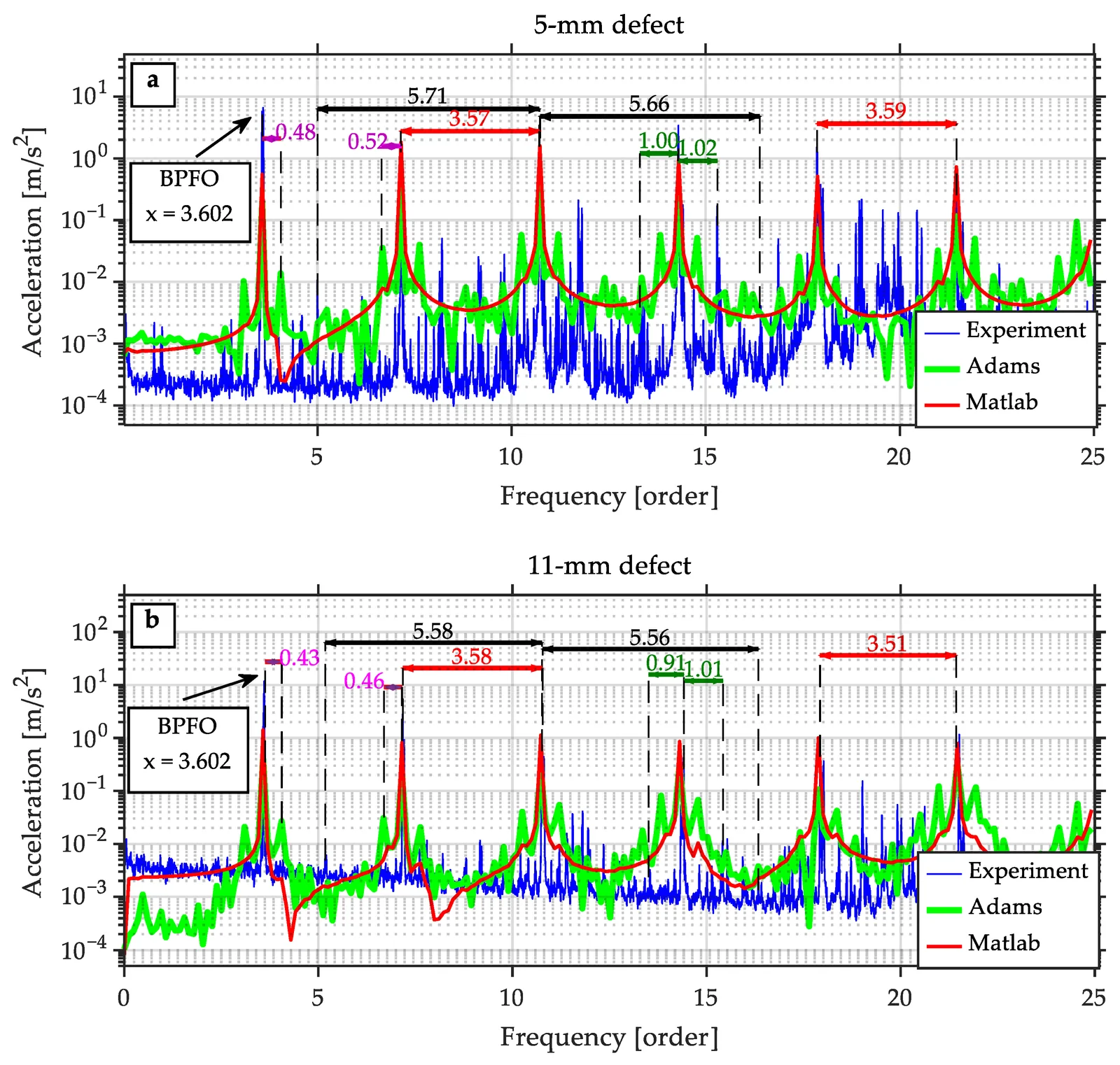
Spectrum of the acceleration signals of 5-mm (**a**) and 11-mm (**b**) outer-race defects. Annotated arrows indicate selected spectral intervals: BPFO spacing (red), BPFI-related spacing (black), shaft-related sidebands (green), and FTF-related sidebands (purple). In the ADAMS spectra, a consistent set of sidebands appears around each BPFO harmonic. These sidebands are marked with purple arrows and measured relative to the first and second BPFO harmonics. The measured intervals are approximately 0.43 to 0.52 orders, while the simulated order resolution is 0.1192 orders. Relative to the analytically predicted fundamental train frequency of 0.40 orders, these intervals correspond to deviations of approximately 0.03–0.12 orders, with the upper end of this range approximately equal to the simulated order resolution. The sideband spacing is therefore consistent with an FTF-related interpretation of this specific modulation component within the ADAMS response, but is treated as a secondary spectral observation rather than as independent validation evidence. To complement the spectra, order-domain spectrograms were generated for the ADAMS model and the experimental data, as shown in Figure 9. Each plot presents defect lengths from 1 mm to 17 mm, in 1-mm increments, along the vertical axis, with order along the horizontal axis. The color scale represents the base-10 logarithm of the acceleration magnitude in m/s^2^. This format enables direct comparison of spectral patterns across a controlled range of defect sizes. To aid visual interpretation, pairs of black dashed lines are overlaid around each analytically predicted BPFO harmonic, with an offset of ±0.2 orders. The BPFO harmonics are clearly resolved across defect sizes in both sources, highlighting the consistency of the modeled spectral response.

This section examines the spectral content of the outer-ring acceleration response across the experiment and the two simulation models. Figure 8 presents order-domain spectra for the 5-mm and 11-mm defect lengths, derived from the experimental measurements, the MATLAB model, and the ADAMS model. Harmonic components associated with the BPFO appear prominently in all spectra as narrow, regularly spaced peaks. These components serve as key indicators of periodic outer-race defect interaction and provide the main reference for evaluating spectral consistency.

To aid interpretation, the figure includes colored arrows marking selected spectral intervals. The red arrows highlight the spacing between selected BPFO harmonics, specifically between the second and third components and between the fifth and sixth components. The corresponding interval values are shown directly in the figure. The first BPFO harmonic is also annotated at an order-domain location of 3.602 for both defect lengths, demonstrating its proximity to the analytically predicted BPFO of 3.59. Although the measured intervals slightly deviate from the ideal value, ranging from 3.51 to 3.59, these differences are consistent with the order resolution of the spectra. The order resolution is 0.1192 for both the MATLAB and ADAMS outputs and 0.0078 for the experimental data. This difference reflects the different sampling densities and FFT lengths used in the simulation and experimental processing paths. The simulations use a high sampling rate and provide fine time resolution to resolve local contact mechanics, but the resulting computational cost restricted the available steady-state record to ten shaft rotations. While generating longer simulation records would allow a larger FFT block and thereby improve the order-domain resolution, this duration proved sufficient to validate the primary periodic structure of the defect response. The experimental data, recorded at 50 kHz over a full minute, were resampled with respect to shaft position and yielded the finer 0.0078-order bin spacing under the experimental FFT settings.

Potential sideband activity was examined on both sides of the third BPFO harmonic. In the experimental spectra, distinct peaks appear at offsets close to the expected BPFI-related spacing. For the 5-mm defect, these features appear at orders 5.66 and 5.71, and for the 11-mm defect at orders 5.56 and 5.58. These intervals are marked with black arrows and are close to the analytical BPFI value of 5.41 orders. The peaks are evident in the experimental measurements but absent in the MATLAB model. In the ADAMS spectra, the same region shows modest spectral broadening, which is more pronounced for the 5-mm defect and less distinct for the 11-mm defect.

Shaft-related sidebands, spaced approximately ±1 order from the BPFO harmonics, are visible in the experimental data and marked by green arrows around the fourth BPFO harmonic. Peaks appear at 1.00 and 1.02 order intervals in the 5-mm case, and at 0.91 and 1.01 in the 11-mm case. These components are typically associated with mechanical imperfections such as shaft misalignment, which are not represented in either simulation. MATLAB omits the shaft entirely, while in ADAMS the shaft is constrained to pure axial rotation and cannot introduce lateral disturbances. Although peaks at similar locations appear in the ADAMS spectra, they are more plausibly interpreted as second harmonics of the ±0.5 order sideband structure discussed below.

**Figure 9:**
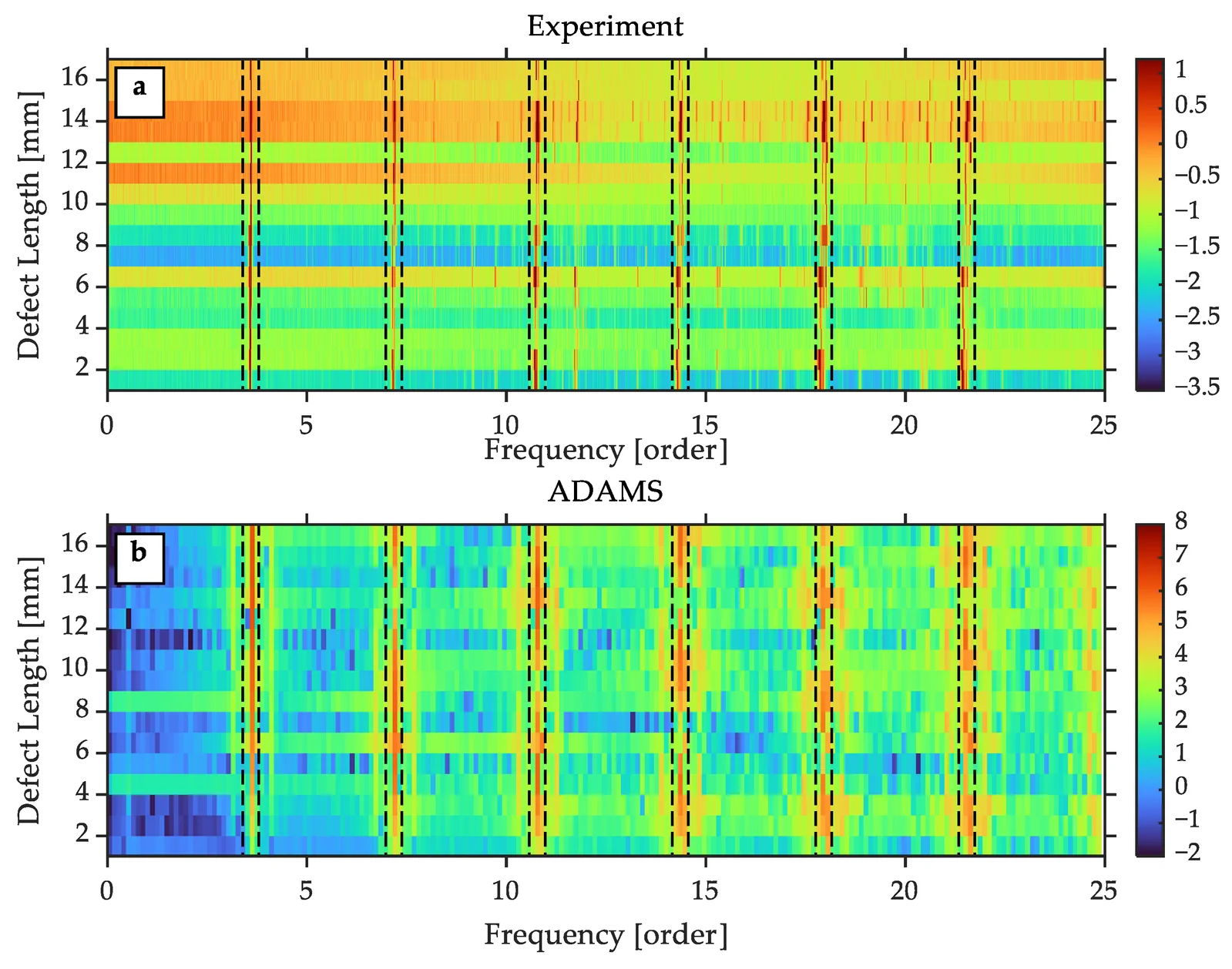
Order-domain spectrograms of outer-ring acceleration for 1–17 mm outer-race defects in (**a**) the experiment and (**b**) the ADAMS simulation. The color scale indicates the log-scaled acceleration magnitude. Dashed lines mark ±0.2-order bands around BPFO harmonics.

The spectrograms and spectral analyses demonstrate that the ADAMS framework successfully captures the primary kinematic and dynamic characteristics of the defective-bearing response across the full range of defect sizes. The consistent tracking of the BPFO harmonic sequence, combined with the presence of modulation effects such as the BPFI-related sidebands, confirms that the model reproduces the essential periodic structure observed in the experimental data. These findings validate the spectral component of the simulation environment and establish its suitability for interpreting complex dynamic interactions in localized bearing faults.

## 5. Discussion

The validation results support the use of the ADAMS model as a mechanically interpretable framework for defective-bearing dynamics. The model reproduces the main order-domain structure of the defective-bearing response, including the BPFO-related harmonic sequence and its spacing across defect sizes. Together with the contact-force analysis, these results indicate that the model captures the main timing logic of rolling-element interaction with the defect. This level of agreement is important because the purpose of the model is not only to generate vibration signals, but also to support interpretation of the mechanical events that produce them.

A central outcome of this study is that some modeling choices directly affect the physical meaning of the simulated response. The outer-ring support formulation is one such choice. When support stiffness is added, part of the applied load can be transferred directly to the ground through the support rather than through the rolling contacts. This changes the effective bearing load and makes the predicted contact forces dependent on the chosen support calibration. In the present model, this leakage mechanism is tied to the bearing clearance and to the transition from the initially coaxial geometry of the rings, cage, and ball pitch circle to the loaded operating state. Models that neglect clearance or initialize the components closer to their loaded equilibrium may therefore not exhibit the same support-induced load leakage. For this reason, the damping-only support used here should not be viewed only as a numerical simplification. It is a modeling choice intended to preserve a consistent load path through the bearing and to keep comparisons between defect sizes unaffected by case-specific support retuning. In this formulation, the dampers provide velocity-dependent resistance to outer-ring motion but carry no static load once the relative motion ceases. The damping coefficient was not obtained from measurements of the experimental housing or support, whose structural dynamics are not represented explicitly in the model. It should therefore be interpreted as a parameter of the simplified support, selected from the sensitivity study to provide sufficient damping of the outer-ring response, rather than as a calibrated estimate of the experimental support damping.

The contact-force comparison also shows that the local contact formulation affects the interpretation of defect interaction. Both the MATLAB and ADAMS models reproduce the same general sequence of entry, floor interaction, and exit, but they differ strongly in the magnitude and character of the exit response. This difference is consistent with the different contact formulations used by the two models. The MATLAB formulation applies a single-point Hertzian contact law with a nominal stiffness along the raceway, whereas ADAMS resolves contact through local geometric overlap between faceted surfaces. The resulting ADAMS response is therefore less dominated by a single sharp exit event and provides a more geometry-dependent description of the transition back into the load zone. This finding is important because it shows that contact-force outputs cannot be interpreted independently of the contact model that produced them. This consideration becomes especially important when simulated contact forces are used as inputs for downstream stress, fatigue, or defect-growth analyses, where differences in the predicted force history may propagate into the predicted evolution of the defect.

Absolute acceleration amplitudes should be interpreted within this scope. Across the examined cases, the simulated acceleration magnitudes are much lower than the experimental measurements. Similar discrepancies have been reported in bearing-defect modeling studies, where analytical or numerical amplitudes often require normalization before comparison with experimental data [9], and where differences in defect-induced impulse modeling and the omission of housing and rotor assemblies can affect amplitude agreement [6,14,24,30,41]. In the present work, the likely source of the mismatch is the absence of the full structural transfer path between the bearing and the experimental sensors [42]. The ADAMS model reports local response at the bearing component level, while the experimental accelerometers measure vibration after transmission through the housing and support structure. Therefore, an explicit distinction is made regarding the validation scope: the physical measurements validate the model’s spectral structure, interaction periodicity and entry-to-exit timing, and dynamic trends across defect sizes, whereas absolute vibration amplitudes are not validated due to the omitted transfer path. Nevertheless, this limitation does not invalidate the framework for interpreting contact-force trends and the mechanical consequences of modeling choices. The computational implications of the framework are also relevant for future use. The simulations presented in this paper were performed using the conservative time-step setting originally required by the legacy MATLAB modeling environment. After the ADAMS model was validated, it was found that larger time steps, up to 5 × 10^−5^ s in the examined case, preserve the principal interaction timing and contact-force peak and RMS metrics, while acceleration amplitudes and short-duration local events remain more sensitive. A quantitative time-step sensitivity comparison supporting this observation is provided in Appendix F. This suggests that the framework may be more computationally scalable than the settings used for the present validation campaign indicate. This point should be treated as a practical implication rather than as a separate validation result: the time-step requirement should still be checked when the framework is applied to other bearing geometries, defect sizes, operating speeds, or output quantities. When larger time-step settings are used, ADAMS may introduce additional solver time points beyond the nominal output step sequence. Therefore, subsequent signal processing should be based on the exported time stamps rather than on the step index, and resampling should be applied when subsequent processing requires a uniformly sampled time series. The model should therefore be used within clear boundaries. It is well suited for controlled studies of defect interaction, contact-force behavior, support and load-path effects, and spectral features associated with periodic bearing faults. It can also support future diagnostic or prognostic studies where physically grounded simulated data are useful. At the same time, the present model is not a full machine-structure model, and it should not be used as a direct predictor of experimental acceleration amplitudes without additional housing and transfer-path modeling. Secondary spectral features, such as the half-order sidebands discussed above, are treated as qualitative model characteristics rather than as primary evidence for validation.

The experimental validation presented here focuses on an SKF 6208 deep-groove ball bearing under a benchmark operational regime (40 RPS, 200 N radial load, and an outer-race defect). Beyond this physical baseline, the simulation architecture operates through dedicated, automated multibody modeling pipelines already established for both deep-groove (DG) and angular contact (AC) bearing families (the external scripting framework of which lies outside the scope of this paper). In this environment, a distinct multibody model instance is automatically constructed for each individual simulation run. This enables direct parametric control over operational conditions (rotational speed and multi-axis load vectors) and fault definitions (outer-ring or inner-ring defect locations, length, and depth). Rolling-element defects under combined radial and axial loading have already been demonstrated within this architecture for AC bearings in [36]. Furthermore, scaling to alternative bearing specifications within the DG and AC families is automated: an integrated replication system takes user-defined geometric dimensions and generates the corresponding modeling pipeline.

The structural boundary of the current automated framework lies in transitioning to fundamentally different bearing classes, such as cylindrical roller bearings. Extending the methodology to such architectures requires a one-time manual adaptation of the baseline geometry and contact definitions using the existing framework as a foundation, after which the automated execution workflow applies directly.

Overall, within the scope examined here, the results establish the ADAMS framework as a validated and interpretable simulation environment for localized bearing-defect studies. The main value of the model lies in the combination of observable vibration agreement and access to internal dynamic quantities that are difficult to measure experimentally. The study also shows that the validity of such a framework depends not only on matching diagnostic signatures, but also on whether the support architecture and contact formulation preserve a physically meaningful representation of defect interaction.

## 6. Conclusions

This paper presented and validated a reconfigurable MSC ADAMS framework for simulating localized defects in rolling-element bearings. The model was developed to reproduce observable bearing-fault response while also providing access to internal dynamic quantities, particularly contact forces and defect-interaction behavior, that are difficult to obtain experimentally. The validation results show that the ADAMS model reproduces the main dynamic structure of the defective-bearing response, including the BPFO-related spectral organization and the timing of rolling-element interaction with the defect. These results support the use of the model as a mechanically interpretable simulation framework, rather than only as a signal-generation tool.

The study also showed that support modeling has a direct influence on the physical meaning of the simulated contact forces. Introducing support stiffness can create an additional load path to ground and reduce the load transmitted through the rolling contacts. The damping-only support formulation used in this work preserves a more consistent load path through the bearing and avoids case-specific support retuning when comparing different defect configurations. The contact-formulation comparison further showed that the predicted defect-exit response depends strongly on how local contact is modeled. The single-point Hertzian formulation used in the MATLAB reference model produced a sharper and larger exit-force response, while the ADAMS geometry-based contact formulation produced a more distributed interaction. This difference indicates that contact-force outputs should be interpreted together with the assumptions of the contact model that generated them.

A clear distinction is therefore drawn regarding the validation scope: the experimental results validate the model in terms of spectral structure, interaction periodicity, and entry-to-exit timing, and dynamic trends across defect sizes, whereas absolute vibration amplitudes are not validated. Accordingly, the model should not be interpreted as a full machine-structure predictor. Its primary validated utility remains investigating defect-interaction dynamics, contact-force behavior, support and load-path effects, and the mechanical interpretation of bearing-fault signatures. Within these boundaries, the work contributes a validated, configurable ADAMS framework for localized bearing-defect studies that links observable vibration response to internal contact-force dynamics. By showing how support definition, load-path preservation, contact formulation, and geometric discretization affect the interpretation of the simulated response, the framework provides a basis for future diagnostic, prognostic, and mechanistic bearing-fault studies.

## Figures and Tables

**Table 1:** Contact and friction parameters used in the ADAMS model.

Parameter [Unit]	Ball–Raceway	Ball–Cage
Normal contact formulation	Impact	Impact
Stiffness [N/mm^2^]	2.680 × 10^6^	2.662 × 10^5^
Force exponent	2.2	2.2
Damping coefficient [N·s/mm]	0.1	0.1
Penetration depth [mm]	0.01	0.01
Tangential friction formulation	Coulomb	None
Static friction coefficient	0.30	—
Dynamic friction coefficient	0.15	—
Stiction transition velocity [mm/s]	100	—
Friction transition velocity [mm/s]	1000	—

**Table 2:** Embedded degrees of freedom (DOFs) and constraint definitions for the model components.

Component	Constraint Type	Degrees of Freedom (DOFs)
Outer Ring	Translationally damped to ground, with rotations constrained	Translational (X, Y, Z); rotational DOFs suppressed
Inner Ring	Fixed to shaft	None (relative to shaft)
Shaft	Motion driver	Axial rotation
Rolling Elements	Unconstrained	6 DOFs (free body)
Cage	Unconstrained	6 DOFs (free body)

**Table 3:** Comparison of the experimental reference, legacy MATLAB benchmark, and ADAMS model used in the validation framework.

Aspect	Experiment	MATLAB Model	ADAMS Model
Contact representation	Physical rolling contact	Single-point Hertzian formulation; nominal contact parameters	Geometry-based multipoint contact; ADAMS IMPACT formulation
Structural and kinematic representation	Physical bearing, shaft, and cage	Rigid-body model; no explicit shaft or cage; equal angular ball spacing	Rigid multibody model; explicit shaft and cage; balls and cage free in 6 DOFs; shaft constrained to rotation about its axis
Support and load representation	Physical bearing supports; radial load through shaft	Inner and outer rings connected to ground by spring-damper supports	Outer ring connected to ground by translational dampers; radial load applied directly to outer ring
Defect representation	Artificial EDM outer-race defect	Constant-depth spall; quarter-circle entry/exit transitions; arc-length definition	Constant-depth geometric cutout; chord-length definition; faceted surface geometry
Outputs used in this study	Housing acceleration; shaft angular position	Simulated acceleration; ball–raceway contact forces	Outer-ring acceleration; ball–raceway contact forces; shaft-constraint reaction force
Role and limitation	Physical validation reference; no direct contact-force measurement	Legacy numerical benchmark; simplified structural and local-contact representation	Mechanistic multibody model; full housing and structural transfer path not represented

**Table 4:** Definition of force ranges used for calculating mean reaction forces. The ranges were selected to isolate stable interaction zones and exclude transient peaks.

Spring Stiffness [N/mm]	5-mm Defect Low Range [N]	5-mm Defect High Range [N]	11-mm Defect Low Range [N]	11-mm Defect High Range [N]	Healthy [N]
0	115–140	235–275	125–165	265–315	215–255
200	115–135	235–270	125–155	260–320	
1000	105–125	205–255	115–150	240–280	
2500	85–110	165–215	90–120	190–235	
5000	64–76	115–145	70–90	140–160	

**Table 5:** Contact force values during defect interaction phases for 5-mm and 11-mm spalls, comparing MATLAB and ADAMS results.

Measurement Phase	Defect Size [mm]	MATLAB [N]	ADAMS [N]	Ratio
Pre-Interaction Peak	5	165	125	0.75
	11	108	140	1.30
Floor Hit (1st)	5	273	67	0.25
	11	274, 198, 134, 84	67, 39	0.24, 0.20
Exit Max Force	5	976	187	0.19
	11	1210	331	0.27

**Figure A1:**
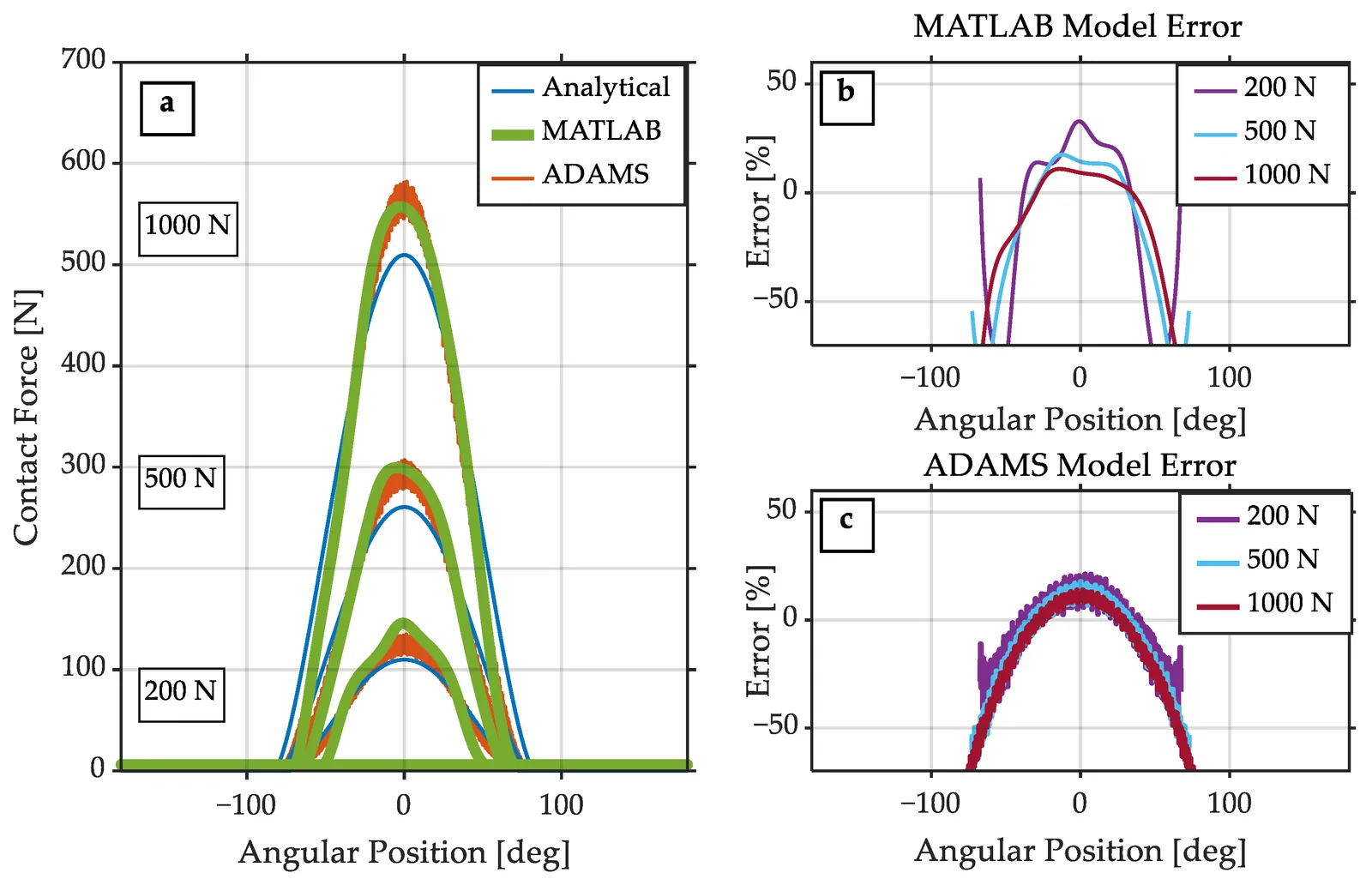
Contact force validation for a healthy deep-groove bearing across simulation and analytical calculations. (**a**) Ball load distribution under three radial loads (200, 500, and 1000 N), comparing ADAMS, MATLAB, and analytical predictions. (**b**) Relative error of MATLAB compared to the analytical reference. (**c**) Relative error of ADAMS compared to the analytical reference.

**Figure A2:**
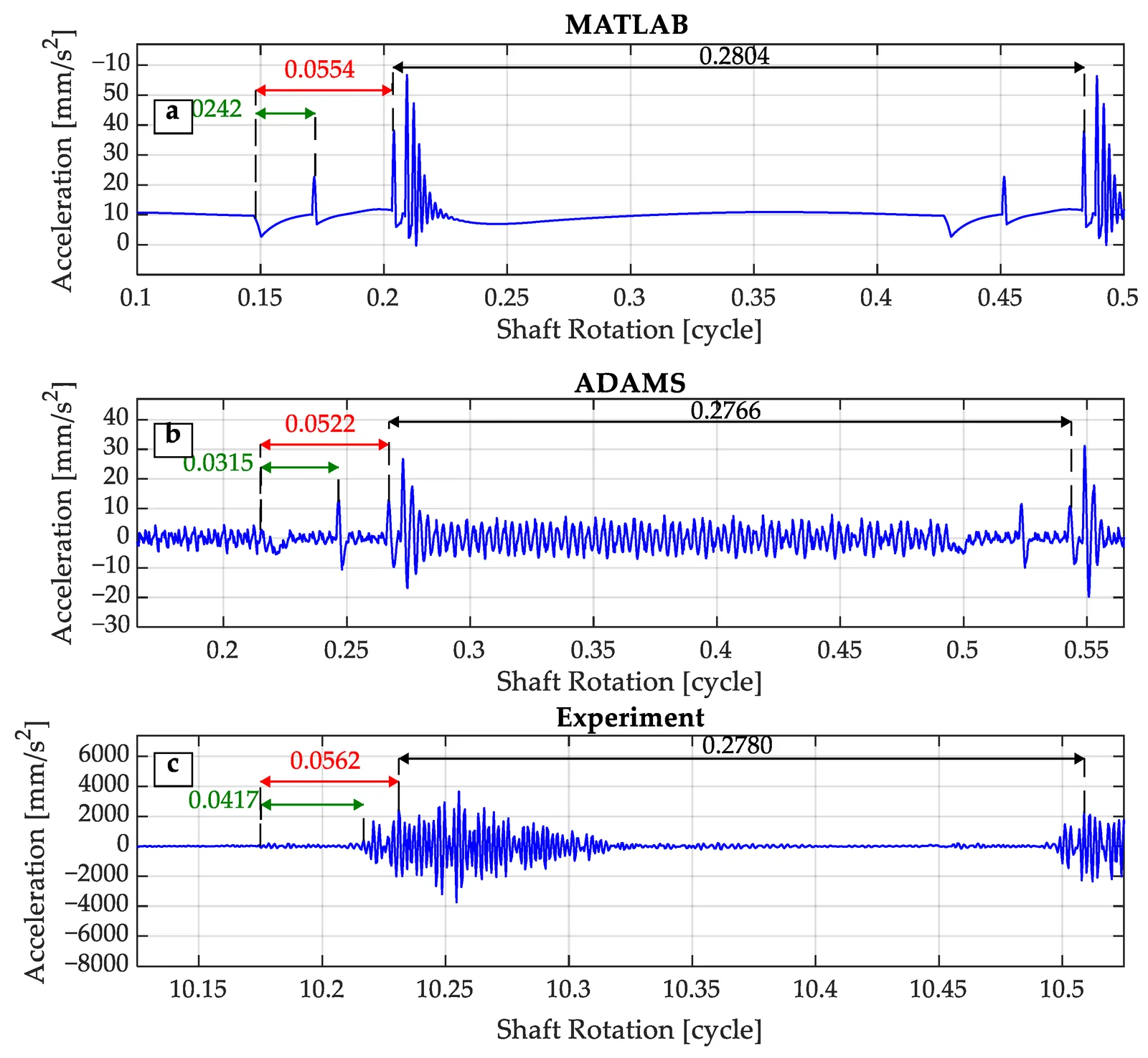
Cycle-domain acceleration signals for a 5-mm outer-race defect, shown for (**a**) MATLAB, (**b**) the ADAMS model, and (**c**) the experiment. Arrows indicate interaction intervals: entry-to-exit (red), entry-to-first floor impact (green), and first exit hit to the next (black).

**Figure A3:**
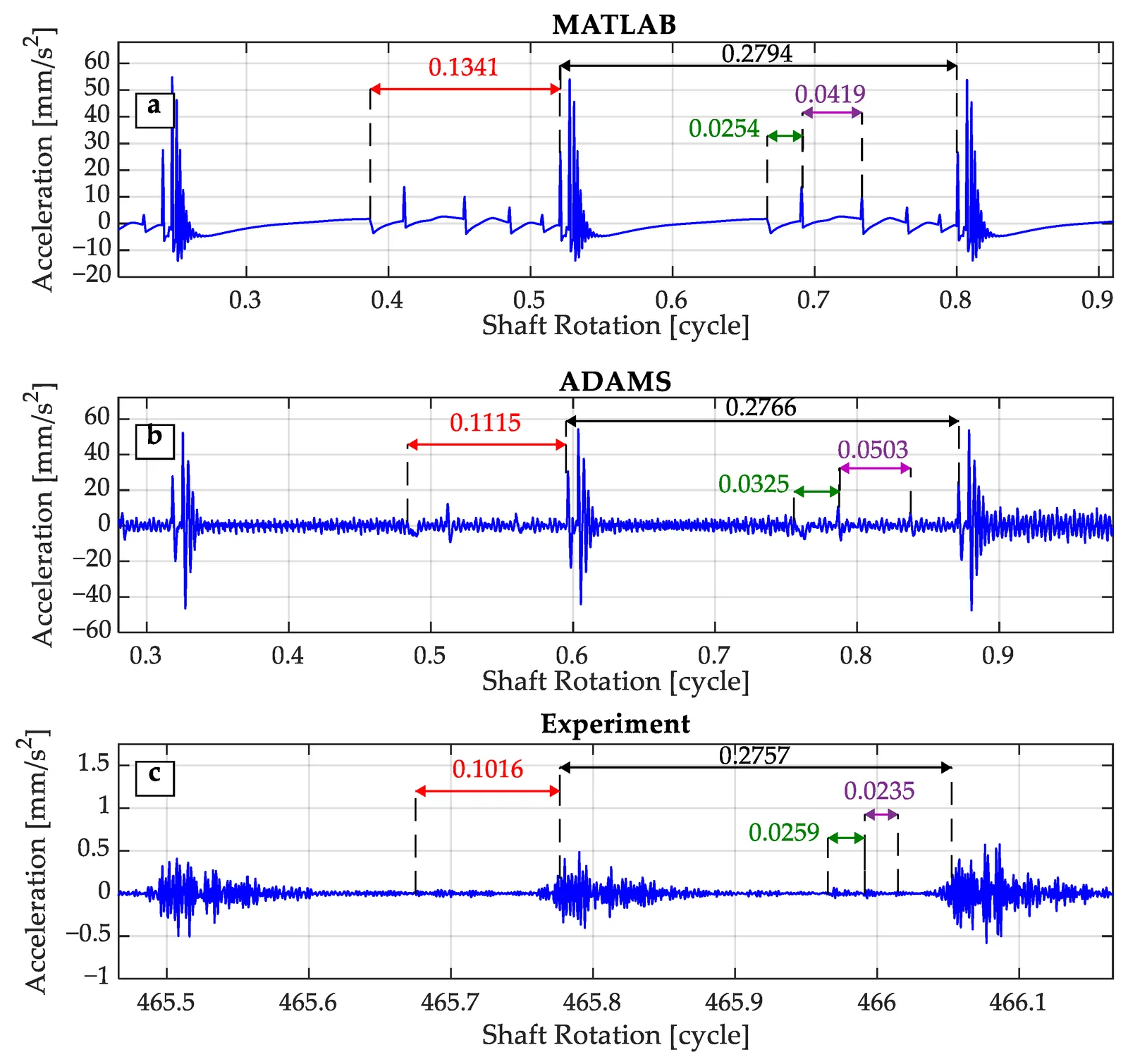
Cycle-domain acceleration signals for an 11-mm outer-race defect, shown for (**a**) MATLAB, (**b**) the ADAMS model, and (**c**) the experiment. Arrows indicate interaction intervals: entry-to-exit (red), entry-to-first floor impact (green), first-to-second floor impact (purple), and first exit hit to the next (black).

**Figure A4:**
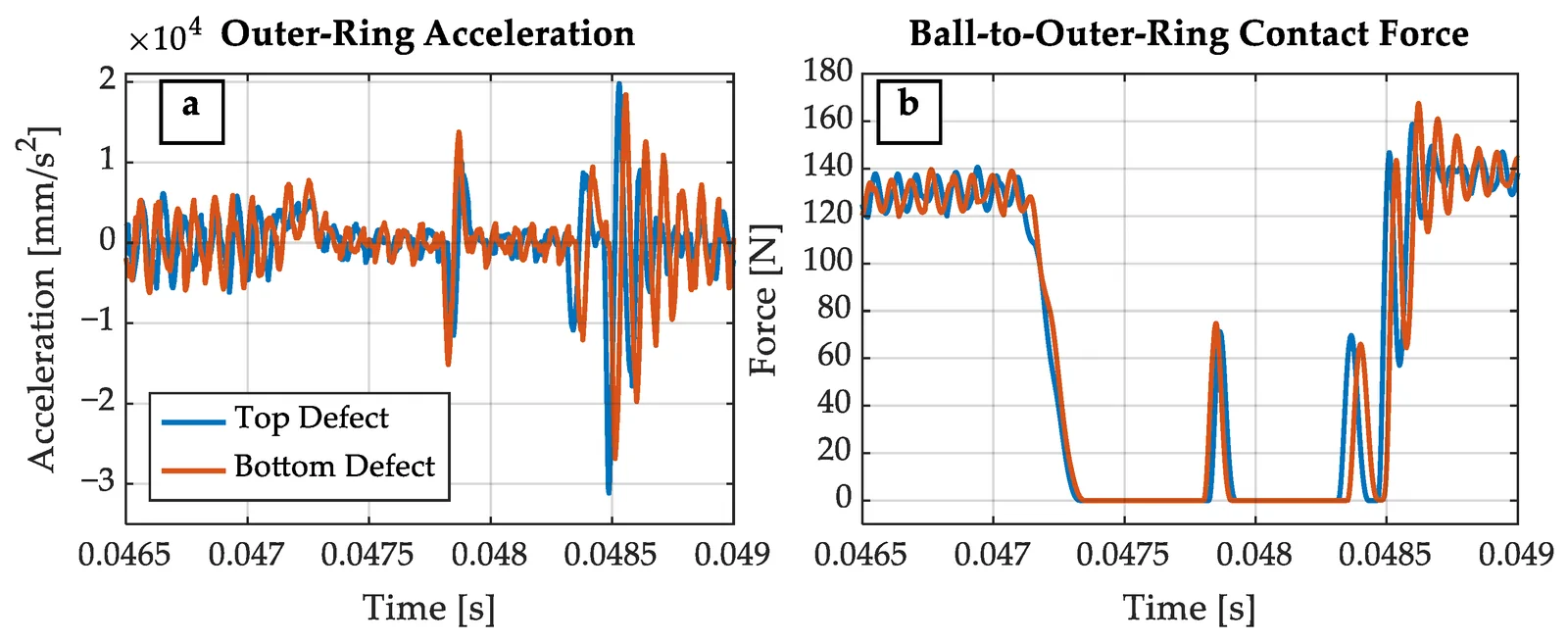
Time-domain comparison of two ADAMS simulations with identical conditions, differing only in the defect position and load direction. Blue: top-defect configuration with outer-ring load. Orange: bottom-defect configuration with inverted load. (**a**) Y-direction acceleration of the outer ring. (**b**) Ball-to-outer-ring contact force.

**Figure A5:**
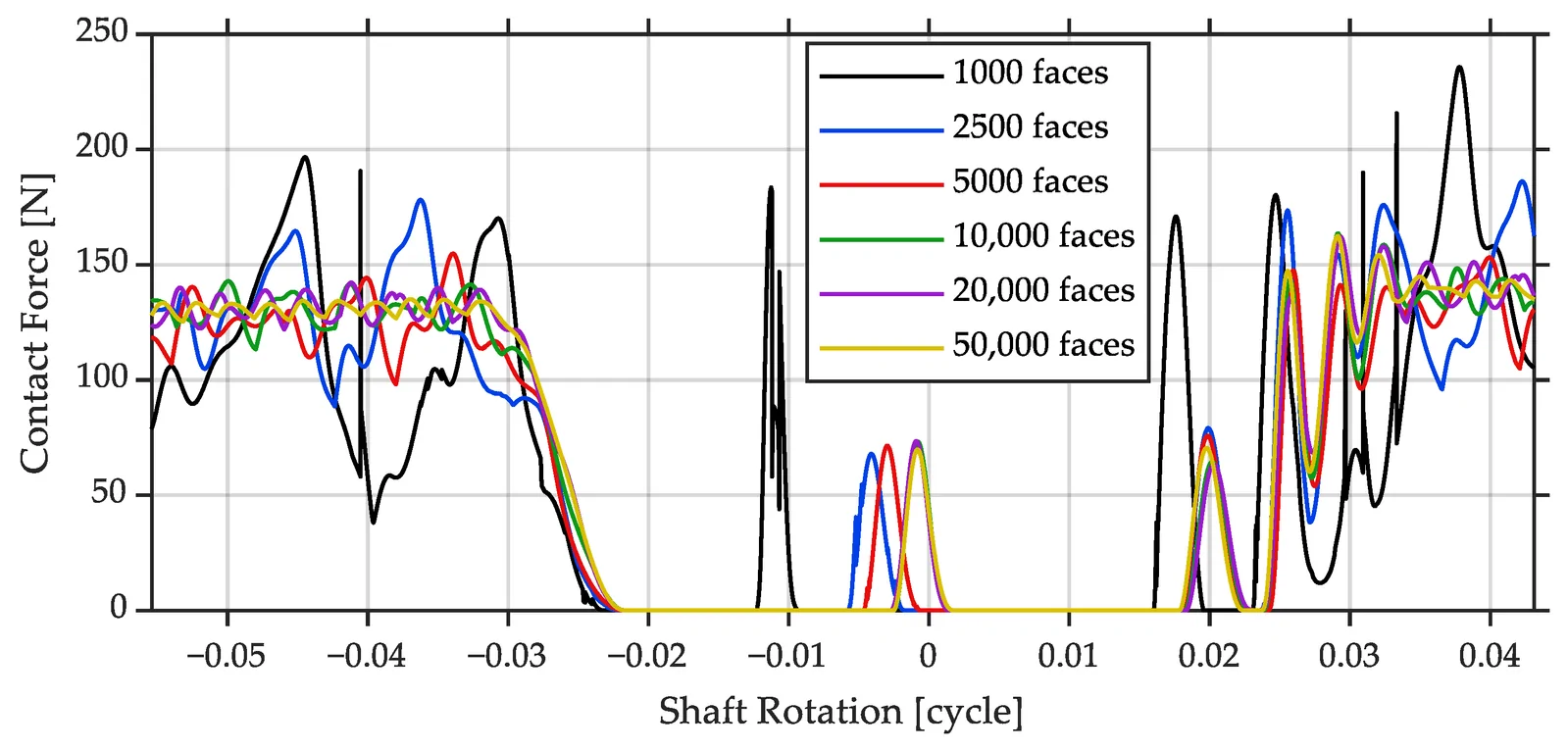
Ball-to-outer-ring contact-force response at different faceting resolutions for the 5-mm outer-ring defect.

**Figure A6:**
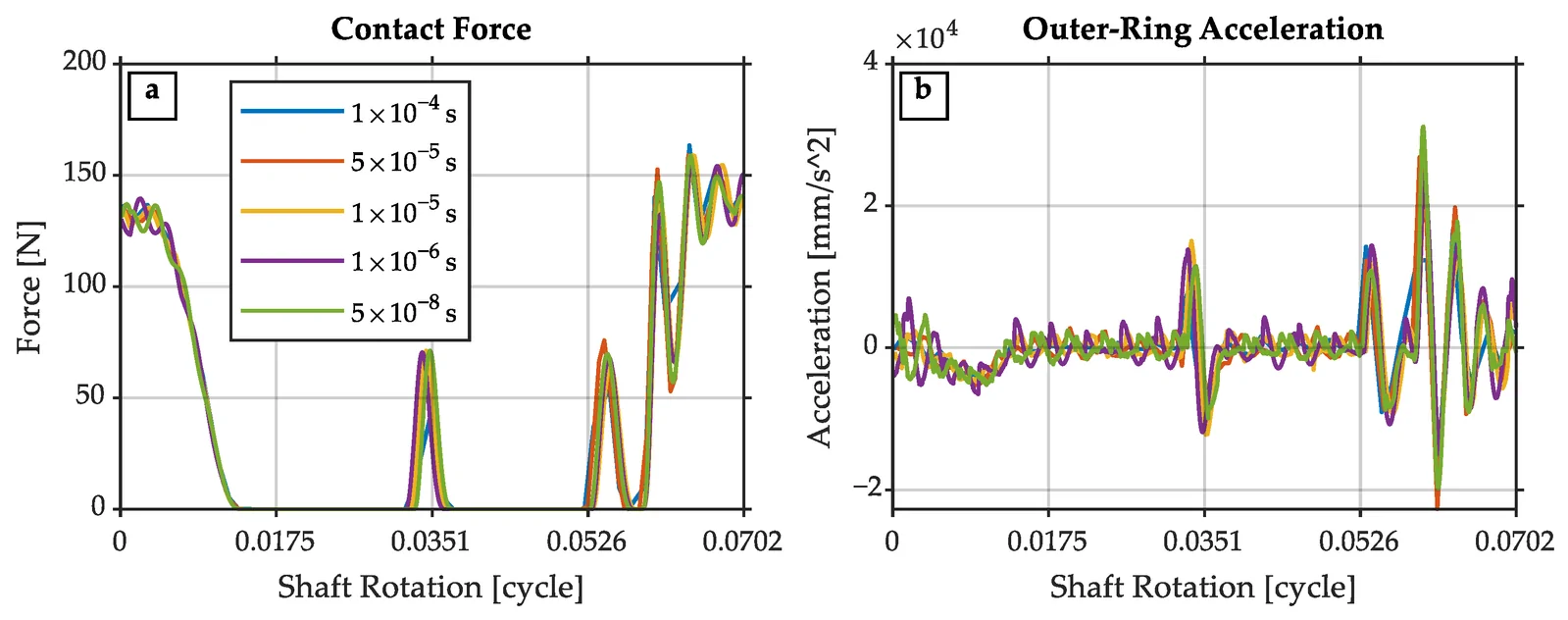
Time-step sensitivity for the 5-mm outer-ring defect case at 40 RPS and under a 200 N radial load: (**a**) ball-to-outer-ring contact force and (**b**) outer-ring acceleration during a defect interaction.

## Data Availability

The data supporting the findings of this study are available from the corresponding author upon reasonable request.

## Appendix A. Healthy-Case Analytical Benchmark

This analysis computes the distribution of contact forces in a deep-groove ball bearing subjected to a static radial load. The formulation follows the method of Harris and Kotzalas [37], using the nonlinear Hertzian contact theory combined with internal radial deflection to model the force on each ball as a function of its angular position. Due to internal clearance, only a subset of the balls are in contact and carry load, and the remainder are unloaded. Each ball transmits force only if the radial deflection is large enough to close the clearance at its angular location. A full derivation of this formulation, along with modeling assumptions, is provided in Appendix B.

Having established the analytical basis, we now evaluate the dynamic model’s predictive accuracy under healthy bearing conditions. Figure A1 compares contact force predictions across the ADAMS simulation, the legacy MATLAB model, and the analytical benchmark using three radial loads: 200 N, 500 N, and 1000 N. In all subplots, the *x*-axis represents the angular position of the rolling element relative to the direction of the applied load; zero degrees corresponds to perfect alignment with the force vector. Figure A1a displays the contact force magnitude predicted by each model, and (b) and (c) present the relative error of the MATLAB and ADAMS models, respectively, compared to the analytical reference. To maintain interpretability, the angular range in error plots is restricted to the contact zone, beyond which the force drops to zero and relative error becomes undefined.

The ADAMS model closely reproduces the analytical contact force profile across all tested loads, with angular spans and force distributions nearly identical to those predicted by theory. The largest positive errors occur near the center of the load zone, where the contact forces are highest, while larger negative relative errors appear toward the edges of the load zone as the analytical contact force decreases. ADAMS maintains a parabolic profile with noise, attributed to surface faceting, while MATLAB shows less smooth relative-error profiles, particularly in the 200 N case. While both ADAMS and MATLAB exhibit elevated error near the edges of the load zone, these regions correspond to minimal contact force and, therefore, exert limited influence on bearing dynamics. Moreover, localized discrepancies in these regions are expected, as small analytical values amplify relative error numerically. Importantly, the model performs well in the central load-bearing region, where material stresses are highest and defect interactions typically occur, supporting its use in subsequent defect-mode validation.

## Appendix B. Full Analytical Derivation of Healthy Bearing Contacts

References in this appendix to Table 7.1 refer to Table 7.1 in Chapter 7 of Harris and Kotzalas [37], not to a table in the present manuscript. Complete cross-reference mapping between the equations derived in this appendix and their original textbook counterparts is provided in Table A1 at the end of this section.

Although the following derivation involves several nonlinear expressions, its purpose and structure are straightforward. We aim to compute the contact forces *F_ψ_* acting on individual balls in a deep-groove bearing under a static radial load, using the expression given in Equation (A1). This relation involves four parameters: the angular position *ψ*, the peak force *F*_max_, the load-distribution factor *λ*, and the contact exponent *n*. These are determined by geometry, material properties, and the ring’s radial shift Δ*_r_*. While Δ*_r_* is unknown, it is obtained from the static radial equilibrium condition, which balances the applied radial force *F_r_* with the rolling-element contact forces. This equilibrium condition cannot be solved analytically in closed form, because *λ* and *J*(*λ*) both depend on Δ*_r_*. Instead, Δ*_r_* is determined numerically by iterating over trial values until the applied radial load is balanced. Once Δ*_r_* is found, the contact force carried by each ball, *F_ψ_*, can be directly calculated.

The contact force on an individual ball (*F_ψ_*) at a given angular position (*ψ*), measured from the direction of the applied radial load, is determined by an expression derived directly from nonlinear Hertzian contact theory, as presented in Equation (A1):

(A1) $$F_{\psi }=F_{\max}{\left[1-\frac{1}{2\lambda }\left(1-\cos\psi \right)\right]}^{n}$$

In this expression, *F*_max_ denotes the peak contact force, which is specifically observed at *ψ* = 0, the angle aligned with the applied radial load. The exponent *n* is defined as 1.5, corresponding to point-contact conditions. It is crucial to note that this equation is valid solely within the angular load zone, when the term within the square brackets yields a positive value. Beyond this region, no contact takes place, and consequently, the contact force *F_ψ_* is zero.

The load-distribution factor *λ*, defined in Equation (A2), is given by the following:

(A2) $$\lambda =\frac{1}{2}\left(1-\frac{P_{d}}{2\Delta_{r}}\right)$$

This expression is used only when the clearance is closed, when Δ*_r_* > *P_d_*/2. Below this threshold, no contact occurs, and *F_ψ_* = 0. This condition also ensures that the bracket term in Equation (A1) remains non-negative and that *F_ψ_* does not exceed *F*_max_.

The total radial force *F_r_* applied to the bearing must equal the sum of the contact forces exerted by the loaded balls. This condition is expressed in Equation (A3):

(A3) $$F_{r}=Z\cdot F_{\max}\cdot J\left(\lambda \right)$$

Here, *Z* is the number of balls, and *J*(*λ*) is a dimensionless integral factor that accounts for the angular extent of the load zone. Values of *J*(*λ*) are obtained by interpolation from Table 7.1 in Harris and Kotzalas [37]. The peak contact force *F*_max_ is defined in Equation (A4):

(A4) $$F_{\max}=K_{n}{\left(\Delta_{r}-\frac{P_{d}}{2}\right)}^{n}$$

This expression links the ring’s radial shift Δ*_r_* to the maximum force acting on any ball. Once substituted into Equation (A3), it yields a nonlinear equilibrium condition with Δ*_r_* as the only unknown. Since the applied force *F_r_* is known, the equation can be solved numerically by iterating over Δ*_r_*, using the tabulated values of *J*(*λ*) and the definition of *λ* in Equation (A2). This structure makes the problem well-posed and computationally straightforward. The only remaining step is to express the stiffness coefficient *K_eff_*, which incorporates the geometry and material properties.

The stiffness coefficient *K_eff_* is defined in Equation (A5) as a generalized combination of two contact stiffnesses:

(A5) $$K_{eff}={\left(\frac{1}{{\left(1/K_{i}\right)}^{1/n}}+\frac{1}{{\left(1/K_{o}\right)}^{1/n}}\right)}^{n}$$

Here, *K_i_* and *K_o_* are the local stiffness values of the inner and outer bodies, respectively. This formulation allows each side of the contact to be treated independently, if required. In the present analysis, the contact is symmetrical, and both sides are assigned the same local stiffness expression. Therefore, both *K_i_* and *K_o_* are taken as equal to *K_c_*, given in Equation (A6):

(A6) $$K_{c}=2.15\cdot 10^{5}\sum \rho^{-1/2}{\left(\delta^{*}\right)}^{-3/2}$$

Here, Σ*ρ* represents the curvature sum of the contacting surfaces, which is inversely proportional to the local radius of curvature. For this bearing, the raceway and ball curvatures are close in value, and the difference is negligible for the calculation. The coefficient 2.15 × 10^5^ is taken directly from Harris and Kotzalas [37] and is valid for steel-ball-to-steel-raceway contact. The term *δ*^*^ is a dimensional scaling factor used to ensure unit consistency. It is not a physical input or result. Although Harris and Kotzalas [37] do not explicitly define it, the exponent −3/2 corresponds to the contact exponent *n* = 1.5 used in the force relation of Equation (A4). This formulation ensures that the stiffness coefficient *K_c_* has the correct units with the nonlinear force expression of Equation (A4). In the present analysis, *δ*^*^ is taken as 1 mm.

**Table A1:** Correspondence between the present analytical derivation and the source formulations in Harris and Kotzalas [37].

Formulation in Current Manuscript	Corresponding Source Equation in Harris and Kotzalas [37]	Description/Physical Meaning
Equation (A1)	Equation (7.15)	Contact force distribution *F_ψ_* based on angular position
Equation (A2)	Equation (7.12)	Load-distribution factor (clearance ratio) *λ*
Equation (A3)	Equation (7.19)	Static equilibrium for total radial force *F*_r_
Equation (A4)	Equation (7.21)	Peak contact force *F*_max_ as a function of radial shift
Equation (A5)	Equation (7.6)	Effective stiffness coefficient *K_eff_* combination
Equation (A6)	Equation (7.8)	Material and curvature-based contact stiffness *K_c_*

## Appendix C. Cycle-Domain Defect-Interaction Analysis

This section compares acceleration signals from ADAMS, MATLAB, and the experiment for two defect lengths. This comparison provides a useful channel for comparing the timing and sequence of defect-interaction events across the three sources. Figure A2 and Figure A3 present cycle-domain acceleration signals for the 5-mm and 11-mm defect lengths, respectively. Each figure includes three subplots: (a) MATLAB, (b) ADAMS, and (c) the experimental signal. The signals in each figure were temporally shifted to align defect interactions across sources for easier visual comparison. While the *x*-axis range differs between the 5-mm and 11-mm figures, it is kept identical across subplots within each figure. The interaction intervals marked in these figures are summarized in Table A2 and used below for cycle-domain timing comparisons.

Each signal exhibits the expected defect-interaction pattern: an initial entry event, followed by one or more spall floor impacts, and a subsequent exit. These transitions are annotated with colored arrows and numerical values of the measured interval representing cycle intervals between specific events:

- Black: the distance between successive interaction cycles (first exit hit to the next)
- Red: entry to first exit hit
- Green: entry to first floor impact
- Purple: (11 mm only): first to second floor impact

The measured interval between repeated defect interactions (black arrows) serves as a baseline check to verify that both the simulation and measurement exhibit the expected periodicity of outer-race defect interactions. The analytical BPFO for the bearing used in this study, reported in Section 2.5, is 3.59 orders, yielding a predicted interval of 1/BPFO ≈ 0.2786 between each defect interaction. For both defects, the black-arrow intervals in Table A2 show that all sources report values within ±0.003 of the analytically calculated 0.2786-cycle interval.

To provide a geometric reference for the main entry-to-exit timing, the interval between defect entry and exit (red arrows) was related to the known defect length using a simple arc-length approximation. Assuming the ball moves along a circular arc defined by the contact surface in the outer raceway, the relation between the traversed arc length and the cycle-domain interval is given in Equation (A7):

(A7) $$L=r\theta =r\left(2\pi \cdot FTF\cdot \Delta_{cycle}\right)\;\Delta_{cycle}=\frac{L}{2\pi r\cdot FTF}$$

Here, *L* is the defect length, *θ* is the angular displacement of the cage in radians during the entry-to-exit interval, Δ*_cycle_* is the cycle-domain interval between entry and exit, *FTF* (0.40 orders) is the cage frequency, and *r* is the radius of the arc traversed by the contact point. In the simulation, this radius is taken as the sum of the torus radii used to define the outer-ring groove: 6.519 mm (minor) and 29.635 mm (major), yielding *r* = 36.154 mm. In Table A2, this relation is used to calculate the analytical entry-to-exit interval from the nominal defect length.

This estimation assumes that contact occurs along a line connecting the outer-ring center, the ball center, and the contact point on the raceway surface. However, due to the finite size of the rolling element and its radial displacement during defect traversal, the ball may strike the exit boundary before this alignment is fully restored. In such cases, the contact occurs on the side of the ball, ahead of the geometric centerline. As a result, the signal peak associated with the defect exit can appear slightly earlier than the true geometric exit, leading to a shorter measured entry-to-exit interval than the ideal geometric estimate. The red-arrow values in Table A2 are therefore interpreted as timing comparisons relative to this analytical estimate, rather than as independent defect-length measurements.

In the MATLAB signals, the defect entry and exit appear as clear impulses and are easily located. In ADAMS, the exit peaks are also well defined and straightforward to detect. The entry events are a bit more ambiguous, typically marked by a drop in acceleration. This transition is often followed by a slight reduction in high-frequency noise, attributed to the ball entering free flight and temporarily disengaging from contact. Floor impacts in ADAMS are distinct and produce isolated peaks. In the experimental signals, the exit events remain visible as local maxima, while the entry and floor impacts appear as low-amplitude features but can still be identified.

The green and purple intervals in Figure A2 and Figure A3 provide further insight into in-defect dynamics beyond the main entry-to-exit timing. In both simulations, the entry-to-first-floor-impact interval remains similar between the 5-mm and 11-mm defects, whereas in the experimental signal it decreases from 0.0417 cycles to 0.0259 cycles. Because these events have lower amplitudes in the experimental signals and are more difficult to identify consistently than the exit events, this comparison is treated as qualitative. The purple interval, indicating a second floor impact, appears only in the 11-mm case and is observed in all three sources. The corresponding intervals differ between sources, but the common presence of this event supports the interpretation that the longer defect can support multiple floor interactions. Taken together, Table A2 shows close agreement in the repeated interaction and entry-to-exit intervals, whereas individual floor-impact times differ more substantially from the experiment. The quantitative timing validation therefore relies primarily on the first two measures.

**Table A2:** Defect event timing in shaft rotations for 5-mm and 11-mm outer-race spalls. Δ values are absolute differences from the corresponding analytical estimate.

Defect Length	Event Type	Exp	MATLAB	ADAMS	Analytic	Δ_Exp_	Δ_MAT_	Δ_ADAMS_
[mm]		[Cycle]						
5	Exit-to-exit	0.2780	0.2804	0.2766	0.2786	0.0006	0.0018	0.0020
5	Entry-to-exit	0.0562	0.0554	0.0522	0.0550	0.0012	0.0004	0.0028
5	Entry-to-floor	0.0417	0.0242	0.0315	—	—	—	—
11	Exit-to-exit	0.2757	0.2794	0.2766	0.2786	0.0029	0.0008	0.0020
11	Entry-to-exit	0.1016	0.1341	0.1115	0.1211	0.0195	0.0130	0.0096
11	Entry-to-1st floor impact	0.0259	0.0254	0.0325	—	—	—	—
11	1st to 2nd floor impact	0.0235	0.0419	0.0503	—	—	—	—

## Appendix D. Sensitivity of the Dynamic Response to Defect Position and Load Path

This appendix evaluates whether differences in load application and defect positioning between the simulation and experimental configurations affect the dynamic responses used for model validation. As described in Section 2.4.2, the experimental rig applies a downward load via suspended weights attached to the shaft, resulting in force transmission through the inner ring and positioning the defect in the lower, loaded region of the bearing. In contrast, the ADAMS simulation applies the load directly to the outer ring, placing the defect at the top, within the primary load zone. This raises the question of whether the system’s dynamic behavior, particularly that of the interaction between the rolling element and the defect, is sensitive to these differences.

To assess this, two ADAMS simulations were conducted under conditions identical to those described in the main paper. The direction of the applied force was reversed to shift the load zone from the top to the bottom of the bearing, and the defect location was adjusted accordingly to remain within the load zone. All other parameters, material properties, geometry, load magnitude, and solver settings were held constant. Gravity remained fixed in the global downward direction. To enable visual alignment in post-processing, the *y*-axis output of the bottom-loaded configuration was multiplied by –1.

Figure A4 compares the two configurations for a 5-mm defect:

- Outer-ring acceleration in the Y-direction (aligned with the load and gravity).
- Contact force between the rolling element and the outer ring.

The results show that the timing, sequence, and shape of the key dynamic events, including the defect entry, floor impacts, and exit, are effectively identical in both cases. Minor differences in the peak amplitudes are present, but these do not affect the structure or interpretation of the signal. Since the model validation focuses on the timing and structure of these events rather than on the absolute amplitude, the results confirm that the model’s behavior is robust to load path and orientation differences.

Only the Y-direction components are shown, as this axis aligns with the applied load and captures the dominant response induced by the defect interactions. These findings support the validity of the simulations presented in the main body of the paper. The mismatch in load application and defect positioning between the simulation and experiment does not compromise the fidelity of the dynamic behavior used in the model validation.

## Appendix E. Geometric-Discretization Sensitivity of Contact Forces

The interpretation of the defect-exit response in Section 3.2 is based on a fundamental difference between the two contact representations. The MATLAB model applies a single-point Hertzian formulation using nominal contact parameters, whereas ADAMS resolves contact through the local geometry of faceted surfaces. Because the ADAMS geometry is discretized, the characteristic difference between the two model responses could be influenced by the selected faceting resolution. A sensitivity analysis was therefore performed to determine how strongly the principal defect-interaction forces depend on this discretization and whether the characteristic distinction between the ADAMS and MATLAB defect-exit responses is retained when the ADAMS surface resolution is changed substantially.

The 5-mm outer-ring defect case at 40 RPS and under a 200 N radial load was evaluated in a dedicated sensitivity series using surface resolutions of 1000, 2500, 5000, 10,000, 20,000, and 50,000 faces. All other model and solver settings were kept unchanged. Figure A5 compares the ball-to-outer-ring contact-force response during a defect interaction. The principal interaction sequence is retained across the examined resolutions: loss of contact at defect entry, impact with the defect floor, and re-establishment of contact at the defect exit. The 1000-face case exhibits pronounced discretization effects, including a strongly distorted floor-impact event and a noticeable shift in its occurrence. At 2500 and 5000 faces, the floor impact remains clearly identifiable but shows smaller timing and amplitude differences, while the responses from 10,000 to 50,000 faces are much more closely aligned. Reducing the number of faces does not change the ADAMS contact formulation; it only coarsens the geometric representation on which the same geometry-based multipoint-contact formulation operates. Importantly, throughout the examined range, the characteristic ADAMS defect-exit response remains distinct from the sharp, high-amplitude rebound obtained with the MATLAB single-point Hertzian formulation.

The numerical sensitivity was quantified using quantities corresponding to the interaction phases examined in the main contact-force analysis: the maximum contact force, the pre-interaction peak representing the defect-entry force, the first defect-floor impact, and the maximum force during the exit stage. In addition, the pre-interaction contact-force fluctuations were quantified using the mean peak-to-peak amplitude of three consecutive local oscillations before defect entry. The resulting values are summarized in Table A3. All Table A3 metrics are extracted from the ball-to-outer-ring contact force over the single defect-interaction window shown in Figure A5.

**Table A3:** Ball-to-outer-ring contact-force metrics at different faceting resolutions.

Faceting [Faces]	Maximum Contact Force [N]	Entry Force [N]	First Floor Impact [N]	Exit Maximum Force [N]	Pre-Interaction Fluctuation [N]
1000	235.76	169.76	182.53	235.72	148.92
2500	186.21	178.12	67.93	186.20	61.51
5000	161.41	154.80	71.38	147.57	35.04
10,000	163.64	141.30	73.01	163.64	18.34
20,000	162.22	139.03	73.46	161.87	14.14
50,000	162.48	133.98	68.53	162.41	6.46

The values in Table A3 were extracted consistently from the ball-to-outer-ring contact force within this dedicated sensitivity series; they are therefore not intended as a direct numerical re-tabulation of Table 5. Table 5 summarizes selected interaction values from the inner- and outer-ring contact-force responses shown in Figure 7; in particular, for the 5-mm case, the 187 N exit maximum reported there corresponds to the stronger ball-to-inner-ring contact response, whereas the exit values in Table A3 refer only to the outer-ring contact. Small differences in the local outer-ring peak values relative to the original analysis are also consistent with the discretization sensitivity examined here, since the faceted representation introduces local contact-force fluctuations and makes peak magnitudes sensitive to the local contact geometry.

The quantitative results show the strongest discretization sensitivity at the coarsest resolutions. At 1000 faces, the maximum contact force reaches 235.76 N, and the mean pre-interaction fluctuation is 148.92 N. With increasing resolution, the principal force quantities become substantially more stable. From 10,000 to 50,000 faces, the maximum contact force remains between 162.22 and 163.64 N, the first floor-impact force remains between 68.53 and 73.46 N, and the exit maximum remains between 161.87 and 163.64 N. The pre-interaction fluctuations remain more sensitive to refinement, decreasing from 18.34 N at 10,000 faces to 6.46 N at 50,000 faces.

The sensitivity results distinguish the effects of geometric discretization from the contact-model difference discussed in Section 3.2. Coarsening the faceted geometry has a clear numerical effect on the ADAMS response, as demonstrated by the increasing pre-interaction fluctuations and by the strongly distorted floor-impact event at 1000 faces. The defect-exit behavior, however, retains the same characteristic form over the substantially different faceting resolutions and does not develop the sharp, high-amplitude rebound obtained with the MATLAB model. This distinction is consistent with the underlying formulations: changing the faceting resolution alters the geometric resolution available to the ADAMS contact calculation, but it does not replace its geometry-dependent multipoint-contact formulation with the single-point Hertzian formulation used in MATLAB. The results therefore indicate that faceting resolution affects the numerical roughness and the quantitative details of local impacts. In contrast, the qualitative difference between the ADAMS and MATLAB defect-exit responses is not a consequence of the particular 20,000-face discretization used in the main simulations.

## Appendix F. Time-Step Sensitivity

A time-step sensitivity analysis was performed using the 5-mm outer-ring defect case at 40 RPS and under a 200 N radial load. Five nominal time-step settings were examined: 5 × 10^−8^, 10^−6^, 10^−5^, 5 × 10^−5^, and 10^−4^ s. Figure A6 compares the ball-to-outer-ring contact force and the outer-ring acceleration during a rolling-element interaction with the defect. For the three largest nominal time-step settings used in this comparison (10^−4^, 5 × 10^−5^, and 10^−5^ s), ADAMS introduced additional solver time points, and the exported time vectors were therefore not uniformly spaced at the nominal step size. The recorded time stamps were used directly to construct the cycle-domain signals. For the 10^−6^ and 5 × 10^−8^ s simulations, the output was uniformly spaced and the nominal time step was used directly.

The peak contact force was extracted from the defect-interaction window shown in Figure A6 for all five simulations. Contact-force RMS and acceleration RMS were evaluated over a common nine-revolution steady-state interval, with time weighting used for the nonuniformly spaced results. Figure A6 shows close overall overlap of the contact-force responses across the tested time-step settings. The acceleration responses likewise show close correspondence in the timing and sequence of the principal interaction features, although their amplitudes are more sensitive to the time-step setting.

Table A4 quantifies these trends. Relative to the 5 × 10^−8^ s reference, the 5 × 10^−5^ s case differed by 0.06% in the interaction peak contact force and by only 0.17% in contact-force RMS, while its acceleration RMS was 16.14% lower. At 10^−6^ s, the corresponding peak differed by 2.66%, while contact-force RMS differed by 0.48%. Across all tested settings, contact-force RMS remained within 0.9% of the reference. The 10^−4^ s case was included as a deliberately coarse comparison beyond the 5 × 10^−5^ s setting considered above. At this setting, local differences become more readily visible around the short-duration floor-impact event, where the corresponding peak is less well resolved, although the overall sequence of the defect interaction remains recognizable. Taken together, the graphical and quantitative comparisons indicate that the 5 × 10^−5^ s setting preserves the principal interaction timing, contact-force response, and RMS metrics for the examined configuration. At the same time, acceleration amplitudes and short-duration local events are more sensitive to time-step enlargement.

**Table A4:** Time-step sensitivity metrics for ball-to-outer-ring contact force and outer-ring acceleration. Percentage differences in parentheses are relative to the 5 × 10^−8^ s reference.

Nominal Time Step [s]	Peak Contact Force [N]	Contact-Force RMS [N]	Acceleration RMS [mm/s^2^]
10^−4^	163.35 (+2.87%)	46.358 (−0.86%)	2945.3 (−17.41%)
5 × 10^−5^	158.89 (+0.06%)	46.683 (−0.17%)	2990.3 (−16.14%)
10^−5^	158.94 (+0.09%)	46.744 (−0.04%)	3023.6 (−15.21%)
10^−6^	154.57 (−2.66%)	46.986 (+0.48%)	3359.9 (−5.78%)
5 × 10^−8^	158.80 (reference)	46.761 (reference)	3566.0 (reference)
